# CD4^+^ T Cells in the Blood of MS Patients Respond to Predicted Epitopes From B cell Receptors Found in Spinal Fluid

**DOI:** 10.3389/fimmu.2020.00598

**Published:** 2020-04-09

**Authors:** Rune A. Høglund, Robert D. Bremel, E. Jane Homan, Silje Bøen Torsetnes, Andreas Lossius, Trygve Holmøy

**Affiliations:** ^1^Department of Neurology, Akershus University Hospital, Lørenskog, Norway; ^2^Institute of Clinical Medicine, University of Oslo, Oslo, Norway; ^3^Clinical Molecular Biology (EpiGen), Medical Division, Akershus University Hospital and University of Oslo, Lørenskog, Norway; ^4^ioGenetics LLC, Madison, WI, United States; ^5^Department of Molecular Medicine, Institute of Basic Medical Sciences, University of Oslo, Oslo, Norway

**Keywords:** B cell, T cell, multiple sclerosis, idiotope, T-B collaboration, IGHV, epitope prediction, Th17

## Abstract

B cells are important pathogenic players in multiple sclerosis (MS), but their exact role is not known. We have previously demonstrated that B cells from cerebrospinal fluid (CSF) of MS patients can activate T cells that specifically recognize antigenic determinants (idiotopes) from their B cell receptors (BCRs). The aim of this study was to evaluate whether *in silico* prediction models could identify antigenic idiotopes of immunoglobulin heavy-chain variable (IGHV) transcriptomes in MS patients. We utilized a previously assembled dataset of CSF IGHV repertoires from MS patients. To guide selection of potential antigenic idiotopes, we used *in silico* predicted HLA-DR affinity, endosomal processing, as well as transcript frequency from nine MS patients. Idiotopes with predicted low affinity and low likelihood of cathepsins cleavage were inert controls. Peripheral blood mononuclear cells from these patients were stimulated with the selected idiotope peptides in presence of anti-CD40 for 12 h. T cells were then labeled for activation status with anti-CD154 antibodies and CD3^+^CD4^+^ T cells phenotyped as memory (CD45RO^+^) or naïve (CD45RO^−^), with potential for brain migration (CXCR3 and/or CCR6 expression). Anti-CD14 and -CD8 were utilized to exclude monocytes and CD8^+^ T cells. Unstimulated cells or insulin peptides were negative controls, and EBNA-1 peptides or CD3/CD28 beads were positive controls. The mean proportion of responding memory CD4^+^ T cells from all nine MS patients was significantly higher for idiotope peptides with predicted high HLA-DR affinity and high likelihood of cathepsin cleavage, than toward predicted inert peptides. Responses were mainly observed toward peptides affiliated with the CDR3 region. Activated memory CD4^+^ T cells expressed the chemokine receptor CCR6, affiliated with a Th17 phenotype and allowing passage into the central nervous system (CNS). This *in vitro* study suggests that that antigenic properties of BCR idiotopes can be identified *in silico* using HLA affinity and endosomal processing predictions. It further indicates that MS patients have a memory T cell repertoire capable of recognizing frequent BCR idiotopes found in endogenous CSF, and that these T cells express chemokine receptors allowing them to reach the CSF B cells expressing these idiotopes.

## Introduction

Multiple sclerosis (MS) is a chronic, inflammatory disease, likely initiated or sustained by the adaptive immune system (1). B cells have recently been attributed a particularly important role, as removing these from circulation efficiently dampens inflammation within the central nervous system (CNS) (2–4). The exact role for the B cells is still unclear and could involve antigen presentation, antibody production or cytokine secretion (5). The memory subset of B cells seems to be particularly relevant, as these are targets for depletion, reduction, or inhibition by several effective MS therapeutic agents (6). The fact that central B cell tolerance mechanisms remain functional in MS, in contrast to type 1 diabetes or rheumatoid arthritis, also argues for a particular role for memory B cells (7).

We and others have previously proposed that memory B cells, due to VDJ recombination, nucleotide insertions or deletions, and somatic mutations within the immunoglobulin variable regions, may have B cell receptors (BCRs) that themselves could contain T cell antigens capable of triggering autoimmune diseases (8–12). We have chosen MS as a model disease to study this concept, as the immune response in MS is characterized by compartmentalized and persisting clonal expansion of B cells that secrete immunoglobulin G (IgG) with these characteristics (13, 14). In line with this concept, human leukocyte antigen (HLA)-DR restricted CD4^+^ T cells from the majority of MS patients, but not from controls with other neurological diseases, responded to self-IgG from the cerebrospinal fluid (CSF) (15). Further proof-of-concept studies using cloned B and T cells from the CSF allowed us to demonstrate that MS patients have T cells specific for antigenic determinants within the variable regions of the immunoglobulins (idiotopes), and that these proliferate, secrete cytokines and kill oligodendrocytes upon antigen stimulation (15–18). Such mechanisms may allow B cells of various specificities to receive help from T cells specific for an unlinked antigen, an idiotope, and has been shown in mouse models to induce immunoglobulin class switching and cause production of auto-antibodies triggering auto-immune disease (12, 19, 20). An analogous immune response is the generation of anti-drug antibodies to therapeutic monoclonal antibodies (mAbs), where T cell epitopes were mapped to the variable regions (21–24). Variable region of mAbs may be chimeric or only partly humanized, providing additional potentially immunogenic idiotopes.

Immunosequencing technology has progressed, and the sheer magnitude of potential idiotopes to assess in patients is impossible to perform *in vitro*. We reasoned there are key steps necessary for idiotope-driven T-B collaboration to occur, including successful endolysosomal processing of the BCR, sufficient affinity for HLA class II molecules, and lack of T cell tolerance. *In silico* models based on these assumptions suggest that nearly half of CSF BCR variable regions from MS patients harbor potential antigenic idiotopes (9). These models included prediction of HLA-DR affinities (25, 26), likelihood of endosomal processing by cysteine cathepsins (27, 28) and modeling of tolerance likelihood based on T cell exposed motifs (TCEM) (9, 29). It has previously been suggested that frequently occurring TCEM in variable regions (i.e., germline framework motifs) could be tolerogenic, while rare motifs [i.e., complementarity determining region (CDR) 3 or motifs resulting from mutations] potentially could be stimulatory to T cells (10, 29). Thymocytes could be exposed to frequent immunoglobulin heavy chain variable (IGHV) TCEM in the thymus by thymic B cells (30), or by dendritic cells sampling serum immunoglobulins (31, 32).

The prediction models used to predict cathepsin cleavage, HLA affinity and TCEM of IGHV have been validated *in silico* (25–27, 29), and for cathepsin cleavage also *in vitro* using monoclonal antibodies (28). It has however not been verified whether this or any other *in silico* model actually predicts a repertoire of idiotopes that actually have a corresponding T cell repertoire. As MS is a chronic inflammatory disease of the CNS, we expected that relevant blood T cells have a memory phenotype with capacity to migrate into the CNS. The aim of the present study was to examine whether MS patients do have a repertoire of CD4^+^ T cells that recognize endogenous idiotopes predicted as stimulatory *in silico*, and that are highly transcribed the patient's own CSF. Our results demonstrate how *in silico* methods can guide identification of T cell stimulatory idiotopes and allow future comparisons between patient groups to establish disease specificity.

## Methods

### Patients

In this study, we investigated materials collected previously from nine relapsing-remitting MS (RRMS) patients from whom we have immunosequenced the CSF IGHV repertoire (9), and from whom we had collected peripheral blood mononuclear cells (PBMC) in parallel with the CSF cells. Demographic and disease characteristics are described in Supplementary Table 1. The nine patients had on average 1,079 (*SD* = 1,213) translated IGHV sequences, which comprised 30–45 amino acids covering part of the framework region 3 (FW3), the entire CDR3 and part of FW4 (dataset available at http://doi.org/10.6084/m9.figshare.5035703). No material was available to perform renewed sequencing of the full IGHV and/or light chain regions. All participants provided written informed consent before participating.

### *In silico* Parameters for Predicting Antigenic Properties of IGHV Idiotopes

We utilized the previously assembled CSF IGHV dataset (9, 33, 34) as a source of idiotopes. This dataset containing 9,711 IGHV amino acid sequences was originally prepared using IMGT/V-QUEST (version 3.3.4, reference directory release 201531-2; 35). For computational reasons three amino acids were added at the amino and carboxy end. The sequences were otherwise private to the individual. Splitting all 9,711 IGHV sequences into all possible 15-mers yielded 323,841 potential peptides. After removal of added amino acids 266,143 remained for analysis). To pre-select potential antigenic or inert idiotopes, we utilized three *in silico* parameters, all of which had previously been computed (9). The base methods are elaborated upon in Supplementary Methods and more specifically summarized here. Firstly, the Johnson SI normalized neural net predicted HLA class II affinity was determined, as described in detail previously (9, 25, 26). Secondly, the likelihood of endosomal processing by key cathepsins S, L or B (9, 27, 28) was evaluated by using “fuzzy logic”, as described previously (9). This method accounts for how HLA class II molecules bind peptides of varying lengths, by allowing predicted cleavage outside the bounds of the core 15-mer and lowering predicted cleavage probability cut-offs to increase sensitivity. Thirdly, the occurrence of T cell exposed motifs (TCEM) IIa or IIb within the potential idiotopes was determined (9, 29). TCEM IIa/b are non-linear pentamers within 15-mer peptides (Supplementary Figure 1), and frequency was calculated based on occurrence in two different datasets (29, 35) to account for intra-dataset bias and include TCEM from the full IGHV region. Peptides predicted to have T cell antigenic properties were those with the highest predicted HLA-DR affinities as well as predicted cleavage with any of the cathepsins selected among the patient's own IGHV sequences (*n* = 12,405). The predicted inert idiotope peptides had opposite attributes, also including HLA-DQ and HLA-DP predictions, selected from the full IGHV dataset. Predicted antigenic idiotopes were further split by the frequency class (FC) of TCEM, where those with rare TCEM [high FC, motifs occurring less than once in every 131,072 (2^17^) IGHV or rarer] were believed to have the highest potential of generating a stimulatory response (*n* = 1,342). Lower FC (TCEM occurring in every 128 IGHV or more frequently) implicated a higher likelihood of T cell tolerance (*n* = 1,337). From idiotopes fulfilling these criteria, the ones from the most abundant transcripts in CSF were chosen. Duplicates among the top candidates were not included. In cases without enough sequences fulfilling the criteria, the affinity limit was adjusted until enough sequences could be included (MS-2 and MS-4, tolerogenic peptides only). The formal criteria are given in Table 1.

**Table 1:** Criteria for *in silico* prediction of antigenic properties.

	**Stimulatory idiotopes**	**Tolerogenic idiotopes**	**Inert idiotopes**
Johnson SI normalized predicted HLA affinity	< -1.5^a^^,^ ^b^	< -1.5^a^^,^ ^b^	>1^a^^,^ ^b^^,^ ^c^
Cathepsin cleavage probability	Cleaved by S or L or B (liberal)	Cleaved by S or L or B (liberal)	Neither S nor L nor B (strict)
TCEM IIa/b frequency class^d^	≥17	≤ 7	Both high and low
*N*=	10/patient	5/patient	Same 7 for all

a Patient specific, based on HLA-DR/DQ/DP typing.

b If too few sequences fulfilled all criteria, this was adjusted until enough sequences could be included.

c Selected from the full pool of patient IGHV sequences. The available predicted HLA affinity had to be low for all the patients' HLA class II variants (incl. DQ and DP in addition to DR).

d *T cell exposed motif frequency scale is inverse logarithmic. Frequency class 17 indicates the motif occurs no more frequently than once every 2^17^ IGHV sequence*.

### Idiotope Peptides

The selected idiotope peptides (Supplementary Table 2), were synthesized by Mimotopes (Australia) to a minimum purity of 70%, with an average purity of 90% supplied. Aliquots of 0.1 mg were dissolved separately immediately prior to use in T cell activation assays with either Milli-Q water, 0.1 % acetic acid, 0.1% ammonia, 20% acetonitrile, or 8 % dimethyl formamide to a batch concentration of 800 μM idiotope peptide. The first solvent tried was always Milli-Q water, but if the peptide was not fully solved then either acetic acid or ammonia was added dependent on the predicted chemical property of each peptide. The last resorts to dissolve the peptides were either acetonitrile or dimethyl formamide. Dimethyl-sulfoxide (DMSO) was not used to avoid oxidative loss of cysteine, tryptophan and methionine rich-sequences, which frequently occur in the IGHV regions. Compatibility of all these solvents was verified by sustained responses to (Epstein Barr nuclear antigen (EBNA)-1 peptides and low background activation in assays using PBMC from healthy donors (Supplementary Figure 2).

### T Cell Activation Assay

**Figure 1:**
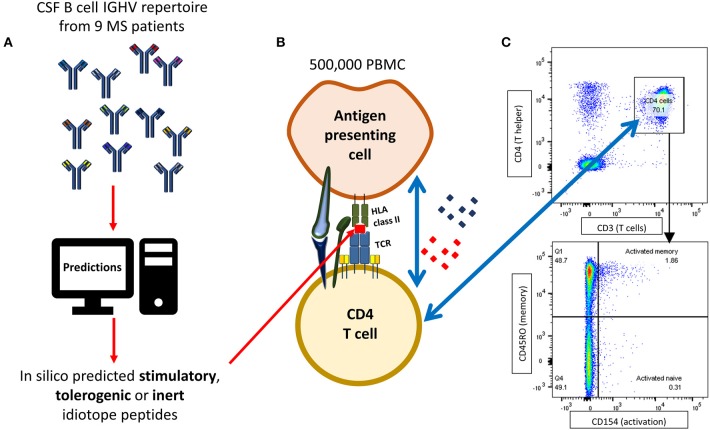
Flow cytometry based idiotope-specific T cell activation assay. **(A)** IGHV amino acid sequences [mean 1,079 (*SD* = 1,213) per patient] from nine MS patients were run through predictive models to identify likely antigenic idiotopes based on HLA class II affinity, cathepsin cleavage and frequency classification (FC) of T cell exposed motifs (TCEM). **(B)** 500,000 PBMC were stimulated with synthetic idiotope peptides predicted to be stimulatory, tolerogenic, or inert as well as positive and negative controls for 12 h in presence of anti-CD40 antibodies. B cells or other professional APCs with idiotope peptides bound to their HLA class II receptor may activate cognate CD4^+^ T cells. **(C)** CD4^+^CD45RO^+^ memory T cells specifically activated by idiotope peptides were detected by surface expression of CD154, upregulated upon TCR stimulation. The example shows a detected memory T cell response to idiotope peptide 12 in patient MS-11.

Activation assays were performed according to previously described and optimized protocols (36, 37), with a few modifications (Figure 1). Cryopreserved PBMC were thawed and immediately centrifuged at 400 × g and washed twice in RPMI 1640 Glutamax (Thermo Fisher Scientific, MA, USA) containing 10% heat inactivated human serum (BioWest, MO, USA). Cells were resuspended to a final concentration of 2.5 × 10^6^ cells/mL, mixed with 1 μg/mL anti-CD40 (blocking antibody, clone G28.5, BioXcell, USA) and plated onto a 96-well U bottom plate. In each well, 500,000 PBMC were stimulated with either 10 μM idiotope peptide (Mimotopes), 1 μg/mL EBNA-1 HLA class II pool (Miltenyi Biotec, Germany), 1 μg/mL insulin peptide pool (Milteny Biotec), 80,000 anti-CD3/CD28 beads (Thermo Fisher Scientific) or an equivalent amount of Milli-Q water. Experiments were performed in technical duplicates. All wells were thoroughly pipette-mixed and incubated for 12 h at 37°C and 5% CO_2_.

### Flow Cytometry

After incubation, cells were transferred to a 96-well V bottom plate, centrifuged at 400 × g and washed with PBS (made in-house). Prior to labeling with antibodies, cells were incubated with fixable Near-IR Live/Dead kit (Thermo Fisher Scientific) to exclude dead cells. After wash, cells were resuspended in PBS with 0.5% FBS (BioRad, Germany) and 2 mM EDTA and labeled according to the manufacturers recommendations with BV421 anti-CD154, FITC anti-CD3, PerCP-Cy5.5 anti-CD4, PE-Cy7 anti-CD45RO, PE anti-CXCR3, and APC anti-CCR6, as well as APC-H7 anti-CD14 and APC-H7 anti-CD8 for dump channel (all BD Biosciences, USA). The fluorochrome panel is described in detail in Supplementary Table 3. After labeling and washing twice, cells were analyzed using a FACS Canto II flow cytometer (BD Biosciences) with a three laser and 4-2-2 detector setup. Compensation was performed using Ultra Comp beads (Thermo Fisher Invitrogen) according to the manufacturer's instructions. Fluorescence minus one (FMO) controls were used to determine gate borders. All reported results are means of technical duplicates. In two cases there were not enough cells to complete all samples (MS-2 and MS-7).

### Statistics

Observed differences in memory T cell activation between each group of idiotope peptides were assessed using a full factorial mixed model with idiotope group as fixed effect and patient subject as random effect to account for interpatient variation in activation. To assess differences in chemokine expression in reactive vs. non-reactive cells in samples with clear T cell responses to idiotope peptide, we applied a full factorial mixed model using activation status as fixed effect and patient subject as random effect. Analysis and all figures were created using JMP® 14 (SAS Institute, USA).

## Results

### Idiotope Peptide Panels

**Figure 2:**
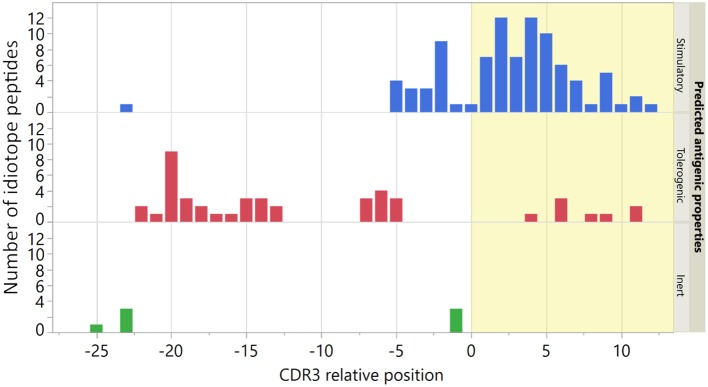
Location of origin for idiotope peptides. The CDR3 relative position was determined by the location of the first amino acid in each 15-mer in the original IGHV sequence. The seven predicted inert peptides were used in all nine patients. Bar colors indicate predicted antigenic properties and yellow shading indicates the CDR3 region.

Demographic and disease characteristics of the nine patients included in the study are shown in Supplementary Table 1. For each patient these we selected a panel of ten predicted stimulatory and five predicted tolerogenic idiotope peptides. Additionally, a common panel of seven predicted inert idiotope peptides was utilized (Supplementary Table 2). We have previously shown that TCEM rarity value in these sequences was associated with peptide locations in IGHV (9, 29). As expected, the predicted stimulatory peptides mapped mainly to CDR3 and the predicted tolerogenic peptides to FW3 (Figure 2). As inert idiotope peptides were selected to have either high or low TCEM frequency, their locations were mixed 3:4 (CDR3:FW3).

### Identification of Idiotope-Specific T Cells

We moved on to identify specific T cell responses. We classified responses three times as high as the same individual's unstimulated control (background) as positive. In all assessed MS patients, we identified robust responses toward CD3/CD28 beads (mean 45% of memory CD4^+^ T cells, range 23–81%, Supplementary Figure 3). As expected, no patient responded toward insulin. Responses toward EBNA-1 peptides varied and were only classified as positive in 3/9 patients, indicating either that they did not have CD4^+^ T cells specific for EBNA-1 or that the assay was incapable of detecting these (Supplementary Figure 3).

The proportion of CD45RO^+^ memory cells among CD4^+^ T cells varied between the MS patients (mean 50%, range 32–68% in unstimulated samples). The patients treated with disease modifying therapies for a longer duration (MS-2 and MS-3), were among those with lowest proportions of memory T cells, potentially affecting our results. To maximize comparability among individuals we therefore chose to use activated cells as a proportion of CD4^+^CD45RO^+^ memory cells for further analysis.

**Figure 3:**
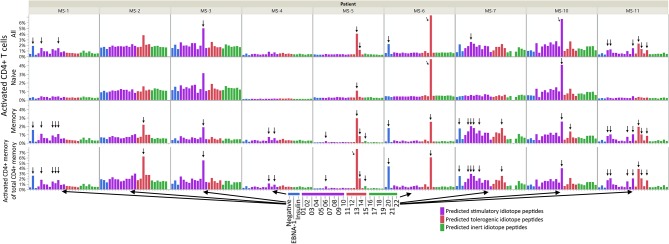
CD4^+^ T cell responses against idiotope peptides. A total of 500,000 PBMC were left unstimulated, or stimulated with EBNA-1 peptide mix, insulin peptide mix, anti CD3/CD28 beads (not shown), or one of 22 idiotope peptides for 12 h in presence of anti-CD40 antibodies and analyzed by flow cytometry. We gated on CD3^+^CD4^+^CD8^−^ T cells and assayed for the activation marker CD154 among all CD4^+^ cells, CD45RO^+^ memory-, or CD45RO^−^ naive cells. Activated cells are presented as proportions of all CD4^+^ cells (upper three panels) or proportion of memory cells (lower panel). Responses were deemed positive (arrows) if the proportion of CD154^+^ cells were 3x higher than in unstimulated (negative) wells.

All patients had idiotope-specific T cells toward at least one predicted antigenic idiotope peptide (Figure 3 and Supplementary Table 4). We observed T cell responses against both predicted stimulatory and tolerogenic peptides, and only one weak response against a predicted inert peptide. Some of the most robust responses were seen in the tolerogenic peptide group. A statistical test using mixed models found mean percentage of activated memory T cells toward predicted stimulatory and tolerogenic idiotopes to be significantly higher than to predicted inert idiotopes [adjusted mean differences 0.22% (CI 0.13–0.30) *p* < 0.0001 and 0.38% (CI 0.18–0.58) *p* = 0.0003, respectively]. There was no significant difference between the predicted stimulatory and tolerogenic peptides (*p* = 0.118). To exclude that our results were results of random activation, we also replicated the experiment in two patients with enough cryopreserved PBMC (MS-7 and MS-11, Supplementary Figure 4), and found comparable responses across two experiments.

**Figure 4:**
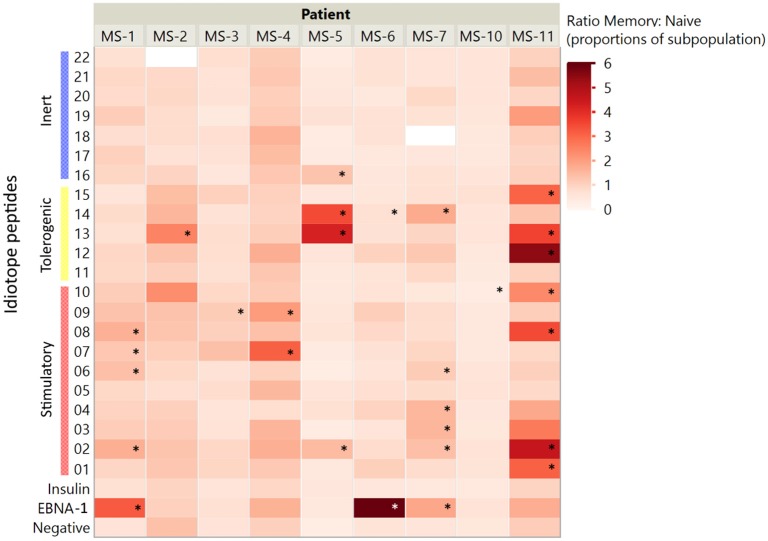
Memory to naïve activation ratios. The ratios of activated CD4^+^ cells (% of CD45RO^+^ memory:% of CD45RO^−^ naïve cells) were assessed for all idiotope peptides. A higher ratio indicates a higher proportion of responder cells among memory CD4^+^ T cells than among naïve cells. The idiotope peptides that elicited memory T cell responses three times higher than the unstimulated control are marked with *.

Responses were not limited to the memory T cells exclusively; in some patients' naïve cells responded toward the same idiotope peptides (Figure 3), possibly indicating a lack of tolerance toward these. Examples included MS-2, MS-3, MS-6, and MS-10, where the first three all had low proportions of memory T cells. We further compared the proportion of activated memory to proportion activated naïve cells (Figure 4). As expected, a higher proportion of responding CD4^+^ T cells were observed in the memory compartment than in the naïve compartment for nearly all antigenic idiotope peptides with observed T cell responses, with the notable exceptions of MS-6 and MS-10.

**Figure 5:**
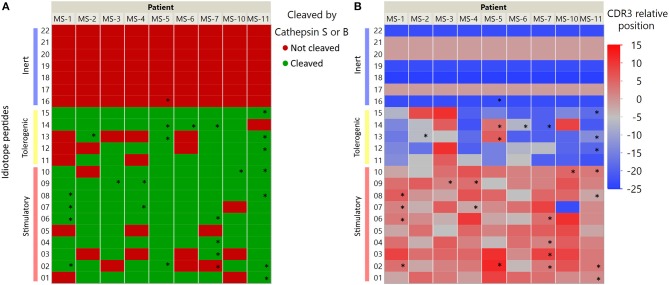
IGHV Localization and predicted possibility of cathepsin processing of antigenic idiotope peptide panel. Idiotope peptides were labeled according to their cathepsin S/B cleavage prediction **(A)** or position of origin within the IGHV sequence **(B)**. Idiotope peptides that generated a memory T cell response are labeled with *.

In order to further characterize the idiotope peptides that elicited CD4^+^ T cell responses, we labeled them using metadata from the IGHV they were derived from and information on cathepsin cleavage prediction (Figure 5). Interestingly, the 24 of 26 idiotope peptides that generated T cell responses were derived from cathepsins S or B cuts, but not from cathepsin L alone. In addition, the idiotope peptides generating responses *in vitro* were most frequently found near the CDR3, regardless of being predicted stimulatory or tolerogenic *in silico*.

### Idiotope-Specific T Cells Are Enriched for CCR6, but Not CXCR3

**Figure 6:**
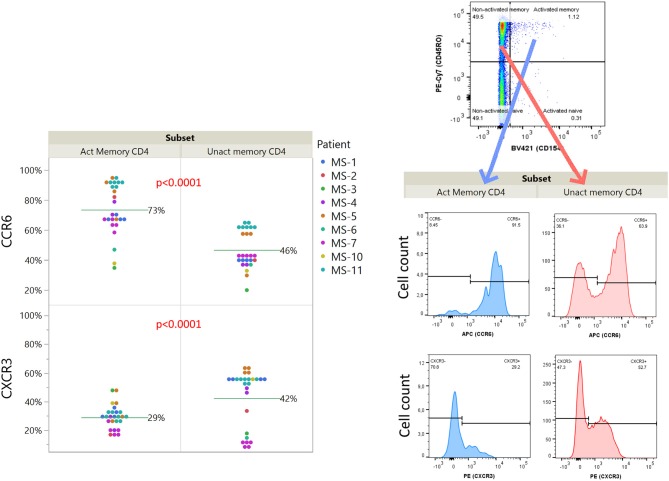
Relative expression of chemokine receptors CCR6 and CXCR3 on idiotope specific CD4^+^ T cells. Expression among activated (CD154^+^) or un-activated (CD154^−^) memory (CD45RO^+^) CD4^+^ T cells responding toward predicted idiotope peptides. *P*-values are results of full factorial mixed model, differences shown are un-adjusted values. An example of gating for analysis (MS-11, sample 15) is shown for illustration.

We further investigated the expression of CCR6 and CXCR3 among idiotope-specific T cells, compared to non-specific cells within the same sample (Figure 6). We found that the idiotope-specific T cells were enriched for CCR6^+^ cells, but not for CXCR3^+^ cells, compatible with at least one mode of entry into CSF of MS patients.

### Antigenic Idiotope Peptides Carry Somatic Mutations

**Figure 7:**
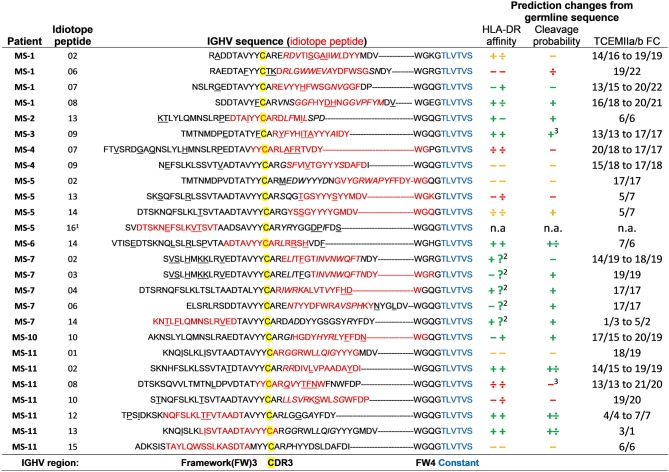
Effect of somatic mutations on predicted HLA DR affinity, cleavage possibility, and frequency classification of T cell exposed motifs. Each idiotope peptide (red) that generated memory T cell responses was aligned within their original IGHV sequence, according to the position of the first cysteine (yellow) of CDR3. Mutations (underlined) and *insertions* (italic) were identified using IMGT V-quest. The IGHV sequences were compared to imputed germline variants to identify changes in predicted outcomes caused by mutations. Change in affinity for patients' two DR alleles was determined by >0.1 difference in ln(IC50) value and >0.1 change in probability for cathepsin S or B cleavage at either side of idiotope peptide ± 3 amino acids. Changes are depicted as + (higher), ÷ lower, and – unchanged. Green indicates imputed net improved-, yellow mixed-, and red indicates net negative effect for antigen presentation. TCEM FC, T cell exposed motif frequency class. ^1^Peptide not in patient's CSF IGHV repertoire. ^2^Missing HLA prediction for one allele. ^3^These also had much lower probability of intra-peptide destruction by cathepsins.

In order to identify mutations within the antigenic idiotope peptides we utilized IMGT V-QUEST (v. 3.5.11, reference directory release 201938-4; 35) to reanalyze the IGHV sequences and identify these characteristics within the idiotope peptides (Figure 7). Not surprisingly, all except two (in MS-11) idiotope peptides carried mutations either within the 15-mer (potentially changing HLA affinity) or in immediate vicinity (potentially affecting cathepsin activities).

To check whether the somatic mutations influenced the predictions for cathepsin cleavage, HLA-DR affinity and TCEM FC, we identified corresponding germline sequences with the closest relative IGH-VDJ genes as given by IMGT/V-QUEST (38) and compared predicted outcomes for mutated and germline sequences. We then ran the *in silico* predictions to assess whether the mutations influenced predicted HLA-DR, probability for cathepsin S or B cleavage or rarity of TCEM of idiotope peptides. The predicted inert peptides were not selected in a patient specific manner and the patient's HLA type could not have contributed to mutation selection, they were therefore left out of this analysis. As expected, mutations were associated with rarer TCEM (higher FC). We found that approximately half of the antigenic peptides carried mutations with net positive effects on the predictions (increased affinity or cleavage likelihood). Six had mixed changes, and five had imputed negative changes for antigen presentation. Note that even the last group of peptides had HLA-DR affinities sufficiently high to be included in the initial analysis of potentially antigenic idiotope peptides, even though the mutations lowered their predicted HLA-DR affinities compared to the corresponding germline encoded sequence.

## Discussion

We have suggested that the inflammation observed in MS can be initiated and/or maintained by idiotope-specific T cells driving an unlinked T-B collaboration within the CNS (8, 18), and that neural network prediction models can identify idiotopes with the requirements needed to be presented to idiotope-specific T cells (9). Here, we demonstrate *in vitro* that MS patients actually have a repertoire of CD4^+^ T cells that recognize highly transcribed idiotopes from endogenous CSF B cells, as predicted *in silico*. We further show that these antigenic idiotopes are associated mainly with the CDR3, predicted cleavage by cathepsins expressed in B cells and with mutations in the IGHV region. Although previous studies using cloned CSF B and T cells provided proof-of-principle that idiotope specific CD4^+^ T cells occur in MS (17, 18), these clones only represent a very small and selected fraction of the repertoires of idiotope-connected T and B cells, and their phenotypes are likely affected by lengthy *in vitro* culture. Our current findings show that the combination of high throughput immunosequencing, *in silico* prediction analysis and *in vitro* stimulation allow quick and extensive identification of highly transcribed antigenic idiotopes and idiotope-specific T cells from unmanipulated PBMC. This paves the way for further characterization of phenotype and disease specificity of idiotope bearing B cells and idiotope specific T cells.

The amount of cryopreserved PBMC collected in parallel with the CSF B cells did not allow further functional studies on cytokine profile, proliferation, and HLA restriction. We cannot, therefore rule out potential non-specific activation entirely, although the overweight of memory responses and the clear difference in responses against idiotope peptides predicted as stimulatory vs. inhibitory indicate otherwise. The lack of PBMC also imposed some other limitations: It precluded experiments on sorted lymphocyte subsets, which could have indicated B cell presentation of idiotope peptides by B cells. Moreover, we were not able to provide replicates of all stimulations including one negative control, which was only performed in monoplicate. The results of the unstimulated wells were however very consistent with those stimulated with insulin peptides or predicted negative idiotope peptides.

In previous work, we identified idiotopes within the IGHV regions generating HLA-DR restricted responses in two MS patients (17, 18). These idiotopes were also associated with mutations and were capable of triggering T cells to destroy oligodendrocytes (16). Development of high throughput sequencing techniques allowed us to assess multiple MS-patient CSF IGHV-repertoires (33), and advances in epitope prediction models including protease cleavage probabilities allowed identification of multiple idiotopes potentially capable of HLA-DR presentation as well as release by cathepsin cleavage (9). In this study, these models could identify idiotopes generating significantly more T cell responses than predicted inert peptides in all patients, but the hit rate was still relatively low given the selection criteria (19% among peptides with predicted high affinity and high probability of cleavage). As we only tested 500,000 PBMC (*n* = 2) for each peptide, our findings nevertheless suggest a high precursor frequency of idiotope-specific CD4^+^ T cells. This is in line with previous observations in MS patients (15, 17, 18). Thus, PBMC from 14 of 21 of MS patients responded toward autologous CSF IgG, whereas only four responded to myelin basic protein (MBP) and five to autologous serum IgG (15). In limiting dilution assays, the frequency of PBMC responding to a DRB1^*^1301-restricted idiotope derived from a mutated IGHV framework region 2 from a CSF B cell clone was 1:2 × 10^4^, while < 1:10^6^ PBMC responded to the corresponding germline-encoded peptide or a MBP peptide (MBP 85–99) suggested to be immunogenic in patients (18). While we were not able to test the HLA restriction of the responding CD4^+^ T cells in this study, the HLA-DR restriction previously shown for CD4^+^ T cell responses both against native self IgG and against idiotope peptides do however fit with the prediction models, as DR alleles exhibited the most consistent affinity pattern for IGHV idiotopes (9). HLA-DRB1^*^15:01 in particular was among those with highest predicted affinity for FW3 and CDR3 derived idiotopes, providing a potential link to inherited risk associated with HLA-DR alleles observed in MS (39). Our results are also in line with the previous observation of several antigenic idiotopes on individual CSF IgG molecules (17).

The high responder rates cause questions as to what is and is not normal in a functional, normal immune system, but our assay was not designed to answer this. We focused our search on the highly transcribed CSF IGHV sequences, and found responding CD4^+^ T cells with potential to reach these, thereby increasing the likelihood of potential for pathogenicity. However, it could very well be similar responses are detectable in in other patient groups. We have previously shown that some patients with other inflammatory diseases also have CD4^+^ T cell responses to self IgG from CSF, although much more rarely than among MS patients (15). T cells specific for auto-antigens are typically rare among CD4^+^ T cells (40), and frequencies of idiotope-specific CD4^+^ cells has not previously been investigated in this assay. Earlier results in proliferation assays indicate they may be more frequent than MBP-specific T cells among in MS patients (15). The number of responder cells in this assay was, although higher than cells responding to EBNA-1 peptides, so low that some variability within replicates was to be expected. While some rare idiotope-specific T cell responses may be undetectable in our experimental setup, it could also indicate the model or selection criteria can be improved. For instance, by including both light and heavy chain variable regions while also considering all cathepsins expressed in B cells simultaneously. Additionally, inclusion of HLA-DQ and -DP could be attempted as well, as -DQ and -DP alleles may have contributed to antigen presentation in these experiments in addition to the predicted role for -DR. Lastly, the IGHV sequences obtained by immunosequencing are rather short and do not include the framework 2 region, where we have previously mapped an antigenic idiotope (18).

We have recently shown that cathepsins S and B, endosomal enzymes expressed in B cells (41), are capable of degrading IgG variable regions into peptides sized to fit HLA class II molecules (28). In B cells with BCR recognizing anti-BCR IgG it was shown that idiotopes from both were presented on major histocompatibility complex (MHC)-II in mice, indicating the BCR and its cognate antigen follow the same pathway of degradation (42). It was thus not surprising to see that 24/25 idiotope peptides with predicted high HLA-DR affinity, and that also elicited memory CD4^+^ T cell responses *in vitro*, were predicted to be released by either cathepsins S or B, but not cathepsin L alone. In the blood, the latter cathepsin is expressed mainly in myeloid APCs (41, 43). We have also observed that B cells from the CSF of MS patients have antigen presenting phenotypes, expressing cathepsins S, H and B, as well as HLA-DR (J. Polak et al., unpublished observations). As a minimal system including cathepsins S, B and H, as well as HLA-DR and -DM was sufficient to generate a diverse HLA class II presented antigen repertoire (44), it is likely these also are key cathepsins in the idiotope-driven response.

There were more idiotope-responses among memory- than naïve T cells. For responding CD4^+^ memory T cells, 21 of 26 idiotope peptides generating responses were associated with the CDR3, consistent with our previous suggestions that this region may be most likely to induce such responses due to the combined events of VDJ-recombination, nucleotide insertions and deletions, and somatic hypermutation (8, 9), but does not exclude possibility of idiotopes generating responses elsewhere in the heavy or light chain variable regions. The presence of idiotope-specific memory T cells indicate previous exposure to idiotopes as antigens, and these cells may gain entry into CSF due to expression of CCR6 (45). This supports a concept of a general dysregulated T-B cell collaboration response in MS, and is in line with current belief that antigen presentation may be a core role for B cells in MS immunopathology (46) and the current knowledge of genetic risk contribution involving antigen presentation (39). However, such a response is not necessarily specific for MS, as a seemingly random mutational activity could generate a similar response in any individual under unfortunate circumstances. For instance, it was shown that B cell presentation of IGHV idiotopes are common occurrences in multiple B cell lymphomas (47, 48), suggesting a potential role in malignant disease. In fact, presentation of BCR idiotopes seems to be a common occurrence upon antigen stimulation in a mouse model (42), and FW3 idiotopes can be eluted from HLA class II from human thymus (49), synovial tissue of rheumatoid- and Lyme arthritis (50), bronchoalveolar lavage samples (51), and in dendritic cells loaded with IvIg (52). The phenomenon is not limited to HLA-DR but was found to occur with HLA-DQ in EBV transformed cell lines as well (53). Further, idiotope-specific T cells have been identified in both SLE (54, 55) and rheumatoid arthritis (56). Thus, although idiotope-specific CD4^+^ T cells seem to be enriched in MS patients compared to controls (15), idiotope-driven T-B collaboration may be a general feature of immune regulation (10, 57).

Some of our observations conflict with previous theories. For instance, it was previously suggested frequently occurring TCEM in variable regions could predict tolerance toward the idiotope (9, 10), as thymocytes would be exposed in the thymus to induce either regulatory T cells (Tregs) or deletion (58). In line with this it has been shown that high concentrations of monoclonal IgG can induce central and peripheral tolerance in various mouse models [reviewed in (10)]. More recently it was shown that repeated exposure to idiotopes caused induction of Foxp3^+^ idiotope-specific T cells in mice (42). However, our observations indicate that at least some of the predicted tolerogenic idiotope peptides had escaped central tolerance mechanisms. In fact, some of the most robust responses were observed toward peptides with frequently occurring TCEM. This observation does not exclude the possibility of peripheral tolerance induction, which potentially could be assessed using proliferation studies or more directly detecting specific regulatory cells. Unfortunately, we did not have access to enough PBMC collected in parallel with the CSF B cells to assess idiotope-specific Tregs, as Tregs are generally very rare and may require enrichment procedures to identify (40, 59). It is possible that TCEM frequency alone is not sufficient to predict tolerance, and that HLA affinity is the chief parameter to assess when searching for idiotope specific T cells. If HLA affinity and TCEM were combined in a single variable (60), a more realistic image of what the T cells are exposed to could be made clear. It has previously been shown that only certain areas of the distinct IGHV families exhibit consistent increase in predicted HLA affinity (9, 29), and several of these seem to correspond to matching increases in probability for cleavage by cathepsins S and B (28). It is also possible that the low frequency or function of Tregs in MS patients (61, 62) contributes to increased immunogenicity of idiotopes, as capacity to suppress is lowered. More recently, it was shown that memory B cells are capable of auto-activating CXCR3^+^CCR6^+^ T cells in an HLA-DR dependent manner. These authors suggested the protein RASGRP2 was responsible (62), but idiotopes could be alternate candidates.

We were not able to directly study intrinsic T-B collaboration. We therefore sought to identify whether MS patients have a repertoire of memory T cells that match endogenous CSF IGHV idiotopes, implying such collaboration had occurred previously and possibly was still ongoing. Previous studies have already established the possibility of direct idiotope-specific T-B collaboration in MS (16, 17). Importantly our study lacked a control group to compare the T cell responses, which could have shed light on disease specificity. A fitting control group for future testing would be patients with other neurological inflammatory disorders, as used in our previous work (9, 15). No cryopreserved PBMC collected in parallel with CSF B cells was available from these patients, who have now been treated for several years with immunosuppressive and immunomodulatory drugs precluding experiments on fresh PBMC.

## Conclusion

By combining high-throughput immunosequencing of CSF B cell repertoires with *in silico* epitope prediction models and *in vitro* activation assays, we were able to identify idiotope-specific memory T cells expressing CCR6 in nine out of nine assessed MS patients. The majority of these idiotope peptides were associated with the CDR3 region, were predicted to have high probability of cleavage by cathepsins S or B and had mutations that influenced affinity or cleavage predictions. This confirms the ability of our predictive models to identify potentially relevant idiotopes, allowing further functional studies in MS patients and relevant controls. It also supports the concept that MS patients have a circulating repertoire of memory T cells capable of invading the CNS, and that cathepsin cleavage plays a role for shaping of the idiotope-specific T cell repertoire.

## Data Availability Statement

The datasets generated for this study are available on request to the corresponding author.

## Ethics Statement

The studies involving human participants were reviewed and approved by Regional Committee for Research Ethics in South-Eastern Norwegian Health Authority (REK Sør-Øst S-04143a). The patients/participants provided their written informed consent to participate in this study.

## Author Contributions

RH contributed with experimental design, performing the experiments, analysis and interpretation of the data, and drafting the manuscript. RB contributed by designing the bioinformatics algorithms, preparing datasets, interpreting the data, and revising the manuscript. EH contributed by interpreting the data and revising the manuscript. ST contributed with experimental design and revised the manuscript. AL contributed by designing the experiments, interpreting the data, and writing the manuscript. TH contributed with conceptualizing the study, interpreting the data, and writing the manuscript.

### Conflict of Interest

RB and EH hold equity in ioGenetics LLC, the company responsible for designing the bioinformatics models used in this project. The remaining authors declare that the research was conducted in the absence of any commercial or financial relationships that could be construed as a potential conflict of interest

## Abbreviations

BCR: B cell receptor
CCR: CC chemokine receptor
CD: cluster of differentiation
CDR: complementarity determining region
CNS: central nervous system
CSF: cerebrospinal fluid
CXCR: CXC chemokine receptor
EBNA: Epstein Barr nuclear antigen
FC: frequency class
FW: framework region
HLA: human leukocyte antigen
IgG: immunoglobulin G
IGHV: immunoglobulin heavy chain variable
MBP: myelin basic protein
MHC: major histocompatibility complex
MS: multiple sclerosis
PBMC: peripheral blood mononuclear cells
TCEM: T cell exposed motif.
