# Supplementary material for: CD4+ T Cells in the Blood of MS Patients Respond to Predicted Epitopes From B cell Receptors Found in Spinal Fluid
Source: Front Immunol. 2020 Apr 9;11:598. doi: 10.3389/fimmu.2020.00598 (PMC7160327; doi:10.3389/fimmu.2020.00598)

Supplementary Material

Supplementary Methods. Neural Networks for MHC binding and biological activity predictions

The IGHV sequences utilized for this paper were previously analysed using *in silico* methods to predict HLA class II affinities and likelihood for cathepsin processing, in addition to calculating frequencies of T cell exposed motifs (TCEM). There were no new predictions or calculations made for this manuscript, except for a limited set of sequences as shown in Figure 8. The neural network prediction algorithms and concepts for prediction of MHC binding used in this study have been described previously (1, 2) but has been updated based on increased size of epitope databases.

The 'Neural' platform in JMP from SAS Institute (3) (Cary, North Carolina) was used for developing the neural networks. This platform embodies a wide variety of neural network (4) development tools and coupled with statistical analytic capabilities. These tools have been combined with the bootstrap aggregation (bagging) concepts developed by Breiman (5) to produce ensembles of neural networks, each of which makes an independent prediction of affinity (as LN (ic_{50})) of a peptide sequence. Neural networks were trained using peptides of 9 amino acids for MHC I and 15 amino acids for MHC II with a single layer perceptron and TanH activation functions. The number of hidden nodes was adjusted to optimize the accuracy of the binding affinity prediction while minimizing overfitting. A perceptron with hidden nodes 3-4 times the length of the training peptide sequence was found to be optimal. Three principal components comprising approximately 90% of the physical property variance was used as input predictors (Method Fig. 1). Ensemble selection is made based on three different statistics (RMSE, SSE and r^2) that capture different features of the predictions.

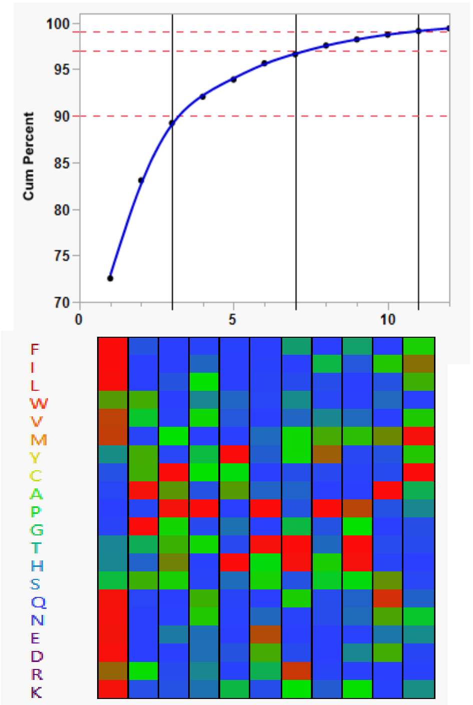

Methods Figure 1 *Increase in cumulative percentage of physical properties variance embodied in increasing principal components and the fractional contribution of each amino acid to each principal component. Higher numbers of principal components produce 5 K-fold fits with lower RMSE but with higher computer run times and possibility for over-fitting. The heat diagram is generated from the squared cosines of the principal components. The squared cosines for each row sum to 1. The red squares indicate the amino acids with the strongest contribution in that dimension.*

Estimates of binding affinity for peptides in primary amino acid sequences of proteins are made by ensembles of 15- 25 neural networks independently derived by the bagging process. For analysing protein sequences, a sliding window indexed by single amino acids is used: 9 amino acids for MHC I and 15 amino acids for MHC II. Use of ensembles makes it possible to predict both the affinity of binding of the peptide to an allele as well as the standard deviations of that binding affinity. The standard deviation of a peptide prediction is related to the root mean square error (RMSE) of the neural network fit of the entire set for an allele.

It has been found that affinity predictions for each allele derived by the neural networks have characteristically different statistical properties (mean, variance, skew). Therefore, to use the predictions the ic50 predictions are transformed to a zero mean, unit variance **distribution** (within protein) using either a SHASH or Johnson

distribution function. For proteomes, or collections of different proteins, the standardization is done within protein. All binding affinities are thus placed on a common scale and can be directly compared in a way that they might be processed in a biological context.

The goal of making these predictions is to provide biological guidance (6). Interpretations of different types of immunological assays are complicated by the inevitable presence of multiple MHC alleles in the same cell and the use of various lengths of peptide in the biologic assays. The clearest, molecularly defined comparison is with tetramer staining of cells and this has been used to derive methods of extrapolation from peptide binding prediction to potential immunological effects. Methods figure 2 shows the comparison of tetramer staining of peptides from influenza HA to a number of different (MHC I and MHC II) alleles as that staining relates to standardized binding affinity predicted by the neural network ensembles based on sliding window predictions of the peptides. Importantly, these peptides have not been 'seen' by the neural networks as they were not in the training sets used to produce the ensembles. Biologic activity curation of the influenza peptides into positive and negative categories was done by IEDB (www.iedb.org). All but four of the MHC II peptides are 20-mers and all the MHC I peptides are 9-mers. For the 20-mers, the predicted highest affinity 15-mer is plotted, as it would be assumed to be the preferred binding register of the longer peptide in the MHC II binding groove. The binding affinities were standardized within the context of their parent HA molecule. There is a very clear difference in the mean affinities in the positive and negative sets. The average negative binding is about 1σ above the mean and the average positive about 1.2σ below the mean. However, there is also an overlap between the two distributions. We also used a recursive partitioning algorithm (sometimes called 'random forest' an unsupervised artificial intelligence technique) to devise criteria for inferring a biologic activity based on the binding data. The dashed lines represent two useful cut points. The line at -0.88 σ represents a point at which false positives are eliminated. The line at +0.34 σ is the optimum separation produced by the recursive partitioning algorithm. At the optimum

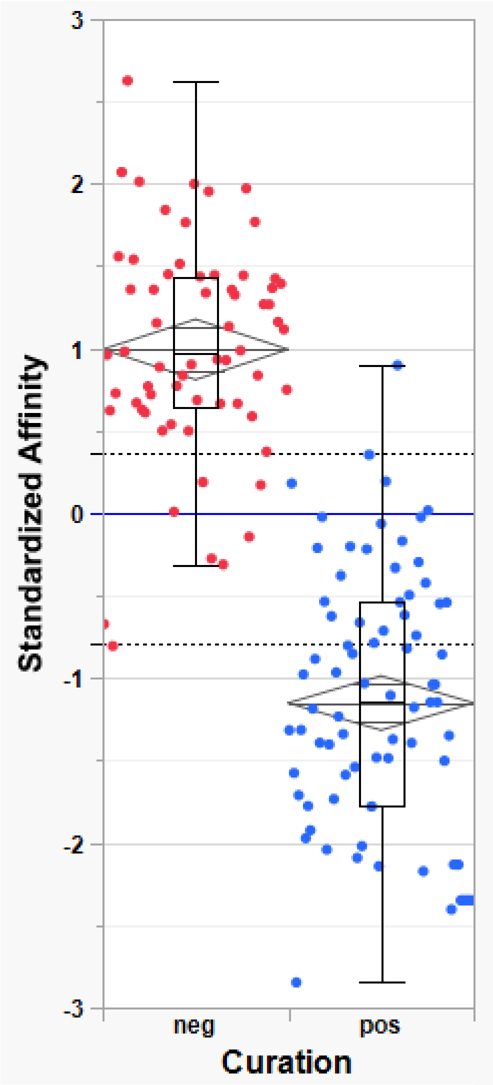

Methods Figure 2 *Staining of MHC tetramers of peptides from influenza HA that are curated as positive or negative by IEDB compared to standardized affinity (in standard deviation units relative to the mean) predicted by the neural network ensembles. The set analysed comprises a total 143 peptides not in the neural network training sets and seven different alleles of MHC II and one MHC I. All MHC I peptides were 9-mers and all but four of the MHC II were 20-mers. For the MHC II peptides the value plotted is the highest affinity 15-mer binding register within the longer peptide. The dashed lines represent optimal cut points determined by a 5 K-fold recursive partitioning. The line at -0.88 σ represents the point which eliminates false negatives and the line at +0.37 σ represents a point of maximal separation of the distributions with a 6.3% misclassification rate that was determined by the recursive partitioning process. Alleles represented (N). B*3501 (10), DRB1*01:01 (10), DRB1*03:01 (2), DRB1*04:01 (73), DRB1*04:04 (15), DRB1*07:01 (16), DRB1*11:01 (16), DRB1*15:01 (1)*

point the misclassification rate is estimated as 6.3%. Based on analysis like these we opted to use -1 σ as an easy to understand point that will eliminate false negatives, and which will include about 60% of true positives. For critical experiments, inclusion of the range of -1 σ → 0 will encompass about 90% of all true positives. This demonstrates an approach to convert primary amino acid sequences into a set of biologically relevant immunological predictions. It has been found that data from other immunological assays such as ELISPOTs is predictable when the combined predictions of all the HLA alleles are used.

REFERENCES

1) Bremel R.D, and Homan E.J, An integrated approach to epitope analysis I: Dimensional reduction, visualization and prediction of MHC binding using amino acid principal components and regression approaches Immunome Research, 2010. **6**:7-22.

2) Bremel R.D, and Homan E.J, An integrated approach to epitope analysis II: A system for proteomic-scale prediction of immunological characteristics. Immunome Research, 2010. **6**:7-22.

3) *Discovering JMP 15*, Copyright 2019 SAS Institute Inc., Cary, NC, USA.

4) Hastie, T., Tibshirani, R., and Friedman, J., 2009, *The Elements of Statistical Learning*. Springer New York.

5) Breiman, L (1996) Bagging predictors, Machine Learning **26**: 123-140.

6) Homan EJ, Bremel RD (2011) Patterns of Predicted T-Cell Epitopes Associated with Antigenic Drift in Influenza H3N2 Hemagglutinin. PLoS ONE 6(10): e26711. doi:10.1371/journal.pone.0026711

Supplementary Table 1. Subject characteristics at time of sample collection

ID	Sex	Age	Diagnosis	Disease duration (months)[a]	Previous or ongoing treatment	Number of relapses	Time since relapse (months)	OCB[b]	CSF cell count[c]	Albumin ratio	IgG index	CSF IGHV Sequences (N)
MS-1	F	29	RR-MS[d]	14	None	1	14	+	3	4	0.85	98
MS-2	F	33	RR-MS	156	IFN β 1a (48 months)	5	60	+	1	5.1	0.81	241
MS-3	F	36	RR-MS	66	GA (27 months)	4	33	+	3	3.8	1.7	417
MS-4	F	63	RR-MS	23	IFN β 1b (2 months)	1	23	+	1	3.6	0.68	109
MS-5	F	27	RR-MS	6	None	4	4	+	2	3.8	1.2	1409
MS-6	F	33	RR-MS[d]	23	None	1	23	+	3	3.5	0.55	3409
MS-7	M	33	RR-MS	48	None	3	3	+	10	17	0.9	2753
MS-10	F	42	RR-MS	35	None	2	18	+	11	3.2	1.06	617
MS-11	F	20	RR-MS	13	None	2	1	+	10[e]	4.8[e]	1[e]	658

F: female; M: male; RR-MS: relapse-remitting multiple sclerosis; OCB: CSF-specific oligoclonal IgG bands; CSF: cerebrospinal fluid; IGHV: Immunoglobulin heavy variable; IFN: interferon; GA: Glatiramer acetate

[a] Since first symptom. [b] OCB positive indicates presence of >2 CSF specific IgG bands on isoelectric focusing. [c] Number of mononuclear cells per microliter CSF.

[d] Patients MS-1 and MS-6 had clinically isolated syndrome at inclusion, but later developed definite RR-MS. [e] Values are from one month prior sampling. The sample from which we acquired cells for sequencing was stopped after 2 mL due to accidental bleeding

Supplementary Table 2. Selected idiotope peptides

Patient	Peptide	Group	Idiotope peptide	Solvent used
MS1	1	Predicted stimulatory	YYCARERDVTISGAI	Acetonitrile
MS1	2	Predicted stimulatory	RDVTISGAIIWLDYY	Ammonia
MS1	3	Predicted stimulatory	SGAIIWLDYYMDVWG	Ammonia
MS1	4	Predicted stimulatory	NERWLRTVAPLDSWG	H_2O
MS1	5	Predicted stimulatory	YYCARFPRAFREDWF	H_2O
MS1	6	Predicted stimulatory	DRLGWWEVAYDFWSG	Ammonia
MS1	7	Predicted stimulatory	REVYYHFWSGNVGGF	Acetic acid
MS1	8	Predicted stimulatory	GGFHYDHNGGVPFYM	H_2O
MS1	9	Predicted stimulatory	ADIVLISAASPFDYW	Ammonia
MS1	10	Predicted stimulatory	YYCARMRGSYRYFFD	H_2O
MS1	11	Predicted tolerogenic	MNSLRAEDTAVYYCA	Ammonia
MS1	12	Predicted tolerogenic	ELRSLRADDTAVYYC	Ammonia
MS1	13	Predicted tolerogenic	YMELRSLRSDDTAVY	Ammonia
MS1	14	Predicted tolerogenic	ELRSLRSDDTAVYYC	H_2O
MS1	15	Predicted tolerogenic	DDTAVYYCATDADIV	Ammonia
MS2	1	Predicted stimulatory	GMSPFYYFYDMDVWG	Ammonia
MS2	2	Predicted stimulatory	YFCARARDYVPFYFG	H_2O
MS2	3	Predicted stimulatory	RDYVPFYFGIEVWGQ	Ammonia
MS2	4	Predicted stimulatory	TYYCGHSLFRDLLSS	H_2O
MS2	5	Predicted stimulatory	RGVVIPMDGGFDYWG	Ammonia
MS2	6	Predicted stimulatory	AKCPHWGNSWYAPFD	H_2O
MS2	7	Predicted stimulatory	LYYCAIAWGFWSTYY	Acetonitrile
MS2	8	Predicted stimulatory	CAIAWGFWSTYYPFY	Acetonitrile
MS2	9	Predicted stimulatory	YEFWSDYYPAPHNWF	Ammonia
MS2	10	Predicted stimulatory	TAVYYCALLRNYYFD	Acetonitrile
MS2	11	Predicted tolerogenic	TAVYYCARLRGVVIP	Acetic acid
MS2	12	Predicted tolerogenic	FFLQWSSLKASDTAM	Acetonitrile
MS2	13	Predicted tolerogenic	DTAIYYCARDLFMIL	Ammonia
MS2	14	Predicted tolerogenic	DTAVYYCAKDLWYYD	Ammonia
MS2	15	Predicted tolerogenic	GWSYYYYYGMDVWGQ	Ammonia
MS3	1	Predicted stimulatory	ASVLWFGVRGSYFDY	Acetonitrile
MS3	2	Predicted stimulatory	RGGNTMVWGLFITSD	Acetonitrile
MS3	3	Predicted stimulatory	NTMVWGLFITSDSYA	Ammonia
MS3	4	Predicted stimulatory	EGFGVIILGPIDYWG	Ammonia
MS3	5	Predicted stimulatory	TATYFCAWTPTAYWR	Acetic acid
MS3	6	Predicted stimulatory	TYFCAWTPTAYWRFE	Acetonitrile
MS3	7	Predicted stimulatory	EGFGVILLGPIDYWG	Ammonia
MS3	8	Predicted stimulatory	YFCARYFYHITAYYY	Acetic acid
MS3	9	Predicted stimulatory	RYFYHITAYYYAIDY	Acetic acid
MS3	10	Predicted stimulatory	FYHITAYYYAIDYWG	DMF
MS3	11	Predicted tolerogenic	LGSQYYYYGMDVWGR	H_2O
MS3	12	Predicted tolerogenic	ICYRYYYYGMDVWGQ	Acetonitrile
MS3	13	Predicted tolerogenic	TAVYYCARPLGRVRG	H_2O

MS3	14	Predicted tolerogenic	LGSHYYYYGMDVWGR	H2O
MS3	15	Predicted tolerogenic	LCYKYYYYGMDVWGQ	Acetonitrile
MS4	1	Predicted stimulatory	YCAREVNLKWELLVF	Acetonitrile
MS4	2	Predicted stimulatory	EVNLKWELLVFDAFD	Ammonia
MS4	3	Predicted stimulatory	NLKWELLVFDAFDIW	Ammonia
MS4	4	Predicted stimulatory	RHREWLRYRGFDYWG	H_2O
MS4	5	Predicted stimulatory	STQLWWGLDFGSWGQ	Ammonia
MS4	6	Predicted stimulatory	LGGVIVPQIVDPWGQ	H_2O
MS4	7	Predicted stimulatory	YYCARLAFRTVDYWG	Acetic acid
MS4	8	Predicted stimulatory	VTYYDILTGFQPKFG	Acetonitrile
MS4	9	Predicted stimulatory	SFVIVTGYYYSDAFD	H_2O
MS4	10	Predicted stimulatory	YYDFWSGPGHIDYWG	Ammonia
MS4	11	Predicted tolerogenic	DTAVYYCATGLFYEI	Ammonia
MS4	12	Predicted tolerogenic	KNSFFLQMNSLRAAD	H_2O
MS4	13	Predicted tolerogenic	QFSLRLSSVTAADTA	Acetonitrile
MS4	14	Predicted tolerogenic	KSKNQFSLKLTSLTA	H_2O
MS4	15	Predicted tolerogenic	KKTLYLQMNSLKTED	H_2O
MS5	1	Predicted stimulatory	YDNGVYGRWAPYFFD	Ammonia
MS5	2	Predicted stimulatory	GVYGRWAPYFFDYWG	Acetonitrile
MS5	3	Predicted stimulatory	PSCYNRNYYFHGLDV	Acetic acid
MS5	4	Predicted stimulatory	AVYYCTTVGHMGYFY	Acetonitrile
MS5	5	Predicted stimulatory	SSEWELMMIVDYWGQ	Ammonia
MS5	6	Predicted stimulatory	YSCARLVIFGMVIID	Acetonitrile
MS5	7	Predicted stimulatory	ARLVIFGMVIIDNVP	DMF
MS5	8	Predicted stimulatory	IFGMVIIDNVPLNWF	Ammonia
MS5	9	Predicted stimulatory	TATFYCAHVWPGYTY	Acetic acid
MS5	10	Predicted stimulatory	WPGYTYGYPNNWLDP	Ammonia
MS5	11	Predicted tolerogenic	LKLRSVTAADTAVYF	Acetic acid
MS5	12	Predicted tolerogenic	LKLRSVTATDTAFYY	H_2O
MS5	13	Predicted tolerogenic	TGSYYYSYYMDVWGK	H_2O
MS5	14	Predicted tolerogenic	YSSGYYYYGMDVWGQ	H_2O
MS5	15	Predicted tolerogenic	LNLRSVTAADTAVYF	Acetonitrile
MS6	1	Predicted stimulatory	SSSLYLYYYSMDVWG	Ammonia
MS6	2	Predicted stimulatory	AMYFCTREGLFPRPF	H_2O
MS6	3	Predicted stimulatory	ARDFYGCRGDKCHLT	H_2O
MS6	4	Predicted stimulatory	PYYYDTTVMDWFDPW	Ammonia
MS6	5	Predicted stimulatory	GLLVLQGWGWAYDYW	Ammonia
MS6	6	Predicted stimulatory	AVYYCVSADTFYYYY	Acetonitrile
MS6	7	Predicted stimulatory	RKFYGAVLQMTFHLW	H_2O
MS6	8	Predicted stimulatory	TAVYYCVCWAGWLVA	Acetonitrile
MS6	9	Predicted stimulatory	RLGYSYGPRWWFDPW	Acetonitrile
MS6	10	Predicted stimulatory	RSEQWLTTTEYFQHW	H_2O
MS6	11	Predicted tolerogenic	TAVYYCARARGWFGL	Acetic acid
MS6	12	Predicted tolerogenic	DDTAVYYCARVWWDQ	Ammonia
MS6	13	Predicted tolerogenic	QNSVYLQMDSLRAED	Ammonia
MS6	14	Predicted tolerogenic	ADTAVYYCARLRRSH	H_2O

MS6	15	Predicted tolerogenic	KNSLYLQMNSLRTED	H_2O
MS7	1	Predicted stimulatory	RGAWLTNDYYTYYGL	H_2O
MS7	2	Predicted stimulatory	ELITFGTINVNWQFT	Ammonia
MS7	3	Predicted stimulatory	TINVNWQFTNDYWGR	Acetonitrile
MS7	4	Predicted stimulatory	RIWRKALVTVYFHDW	H_2O
MS7	5	Predicted stimulatory	YYDFWSGNPDRFDYW	Ammonia
MS7	6	Predicted stimulatory	NTYYDFWRAVSPHKY	H_2O
MS7	7	Predicted stimulatory	GGYIAWGPKKHYYYG	H_2O
MS7	8	Predicted stimulatory	RLPTIWARNPNFHYY	H_2O
MS7	9	Predicted stimulatory	KVADTLAVRLPYFDC	H_2O
MS7	10	Predicted stimulatory	RSHPNFYLGELSFEG	H_2O
MS7	11	Predicted tolerogenic	KNTLYLQMNSLRPED	H_2O
MS7	12	Predicted tolerogenic	DTAIYYCARDRLLWF	Acetonitrile
MS7	13	Predicted tolerogenic	KNTMFLQMNSLRVED	Acetonitrile
MS7	14	Predicted tolerogenic	KNTLFLQMNSLRVED	Acetonitrile
MS7	15	Predicted tolerogenic	KNTLFLQMNSLRAED	H2O
MS10	1	Predicted stimulatory	AKAVRMQLWLFGSWG	Acetonitrile
MS10	2	Predicted stimulatory	NGRFLEWFPLYYFDY	Ammonia
MS10	3	Predicted stimulatory	RFLEWFPLYYFDYWG	Acetonitrile
MS10	4	Predicted stimulatory	NYDILTGFYLASLEL	Ammonia
MS10	5	Predicted stimulatory	DILTGFYLASLELID	Ammonia
MS10	6	Predicted stimulatory	TGFYLASLELIDSWG	Ammonia
MS10	7	Predicted stimulatory	DNAMDILYLQVNSLR	Ammonia
MS10	8	Predicted stimulatory	RINLWTAMPAGGPGL	H_2O
MS10	9	Predicted stimulatory	NLWTAMPAGGPGLND	H_2O
MS10	10	Predicted stimulatory	HGDYHYRLYFFDNWG	Ammonia
MS10	11	Predicted tolerogenic	KSMLYLQMNSLRVED	Acetic acid
MS10	12	Predicted tolerogenic	NSLYLQMDSLRAEDM	Ammonia
MS10	13	Predicted tolerogenic	NSLYLQMNSLRAEDM	Acetonitrile
MS10	14	Predicted tolerogenic	TTSYYYFYYMDVWGK	H_2O
MS10	15	Predicted tolerogenic	KLSFVTAADTAVFYC	Acetonitrile
MS11	1	Predicted stimulatory	ARGGRWLLQIGYYYG	H_2O
MS11	2	Predicted stimulatory	RRDIVLVPAADAYDI	Ammonia
MS11	3	Predicted stimulatory	RLARELILGPEYYYY	Acetonitrile
MS11	4	Predicted stimulatory	RFDIATTVPLGFDYW	Ammonia
MS11	5	Predicted stimulatory	YYCVRLVPKRTATLH	H_2O
MS11	6	Predicted stimulatory	ARVAAWWLAHGTSDS	Acetic acid
MS11	7	Predicted stimulatory	VRDWYRWFGDTGDDY	Ammonia
MS11	8	Predicted stimulatory	YYCARQVYTFNWFNW	Acetic acid
MS11	9	Predicted stimulatory	RQVYTFNWFNWFDPW	Acetonitrile
MS11	10	Predicted stimulatory	LLSVRKSWLSGWFDP	H_2O
MS11	11	Predicted tolerogenic	KSKNQFSLKLTFVTA	H_2O
MS11	12	Predicted tolerogenic	NQFSLKLTFVTAADT	Acetonitrile
MS11	13	Predicted tolerogenic	LISVTAADTAVYYCA	Ammonia
MS11	14	Predicted tolerogenic	AKSLLYLQMNSLRAE	H_2O
MS11	15	Predicted tolerogenic	TAYLQWSSLKASDTA	Acetonitrile

MS10	16	Predicted inert	DTSKNEFSLKVTSVT	H_2O
MS6	17	Predicted inert	FCTRVGDRRHYGGNS	H_2O
MS6	18	Predicted inert	TADKSTRTAYMELSG	H_2O
MS5	19	Predicted inert	DRSKNQFSLKLSSVT	H_2O
MS4	20	Predicted inert	YCARDGRREQLVPNS	H_2O
MS7	21	Predicted inert	YCARDNSNWTRGSGF	H_2O
MS11	22	Predicted inert	DRSKNQFSLKVTSVT	H_2O

Colors correspond to bars in Figure 3

Supplementary Table 3. Surface markers and fluorochromes used on FACS Canto II

Laser	BP Filter	LP	Fluoro-chromes	Marker	Comment	Clone (cat. #)
**407 nm** (Violet)	450/50		**BV421**	**CD154**	T cell activation	TRAP1 (BD 563886)
**488 nm** (Blue)	585/42	556	**PE**	**CXCR3**	Chemokine rec.	11A9 (BD 560619)
	780/60	735	**PE-Cy7**	**CD45RO**	Memory T cells	UCHL1 (BD 337168)
		670 655	**PerCP-Cy5.5**	**CD4**	CD4 T cells	SK3 (BD 566316)
	530/30	502	**FITC**	**CD3**	T cells	UCHT1 (BD 555916)
**633 nm** (Red)	660/20		**APC**	**CCR6**	Chemokine rec.	1C6 (BD 550633)
	780/60	735	**APC-H7**<br><br>**LIVE/DEAD™ Fixable Near-IR**	**CD14**<br>**CD8**	Monocytes<br>CD8 T cells<br>Dead cells (All: dump channel)	MφP9 (BD 560180)<br>SK1 (BD 560179)<br>(L-34959)

BP: Bandpass; LP: longpass

Supplementary Table 4. Mean and standard deviations for T cells activation responses by sample

Group	Samples	N	CD4+ Count	Activated T cells (%memory)		Activated (%CD4)		Activated memory cells (%CD4)		Activated naive cells (%CD4)	
			Mean	Mean	SD	Mean	SD	Mean	SD	Mean	SD
MS-1	Unstimulated	2	19265.5	0.38	0.03	0.47	0.04	0.23	0.02	0.24	0.01
	EBNA	2	19825	**2.60**	1.52	1.88	1.06	1.56	0.92	0.32	0.14
	Insulin	2	17226	0.26	0.02	0.31	0.02	0.16	0.01	0.15	0.01
	CD3/CD28	2	17589	34.68	0.79	30.43	0.98	21.00	0.85	9.43	0.13
	Sample 01	2	18063	0.56	0.05	0.58	0.01	0.34	0.03	0.24	0.02
	Sample 02	2	18521.5	**1.83**	0.37	1.52	0.21	1.10	0.20	0.42	0.01
	Sample 03	2	18752	0.98	0.47	0.94	0.29	0.60	0.30	0.34	0.01
	Sample 04	2	19877.5	0.92	0.12	0.91	0.04	0.56	0.07	0.35	0.03
	Sample 05	2	18313	0.49	0.11	0.51	0.11	0.29	0.07	0.22	0.04
	Sample 06	2	19957.5	**1.48**	0.16	1.31	0.00	0.90	0.09	0.42	0.09
	Sample 07	2	19645	**1.17**	0.10	1.08	0.11	0.71	0.05	0.37	0.06
	Sample 08	2	18583	**1.76**	0.16	1.48	0.08	1.07	0.09	0.42	0.01
	Sample 09	2	19246	0.84	0.08	0.76	0.04	0.51	0.04	0.25	0.01
	Sample 10	2	18169.5	0.95	0.24	0.91	0.13	0.56	0.15	0.35	0.01
	Sample 11	2	19092	0.66	0.07	0.68	0.06	0.40	0.05	0.29	0.01
	Sample 12	2	18933.5	0.62	0.01	0.64	0.01	0.37	0.00	0.27	0.01
	Sample 13	2	18772.5	0.55	0.11	0.63	0.02	0.33	0.07	0.30	0.05
	Sample 14	2	19097.5	0.50	0.09	0.54	0.06	0.30	0.06	0.24	0.00
	Sample 15	2	19226	0.40	0.00	0.53	0.01	0.24	0.00	0.29	0.01
	Sample 16	2	19238	0.68	0.03	0.70	0.02	0.41	0.03	0.29	0.05
	Sample 17	2	18454.5	1.06	0.47	1.04	0.37	0.64	0.28	0.40	0.08
	Sample 18	2	17266.5	0.61	0.13	0.68	0.13	0.37	0.08	0.31	0.06
	Sample 19	2	18880	0.96	0.14	0.91	0.14	0.58	0.08	0.33	0.06
	Sample 20	2	18193	0.55	0.02	0.60	0.04	0.33	0.01	0.27	0.05
	Sample 21	2	17786.5	0.48	0.01	0.50	0.03	0.29	0.01	0.22	0.02
	Sample 22	2	17230	0.48	0.09	0.56	0.04	0.29	0.06	0.27	0.02
MS-2	Unstimulated	1	19635	1.92		1.58		0.68		0.90	
	EBNA	2	19768	1.77	0.02	1.71	0.03	0.64	0.01	1.07	0.01
	Insulin	2	19679.5	1.34	0.07	1.34	0.04	0.47	0.02	0.88	0.02
	CD3/CD28	1	4025	81.12		88.80		20.80		68.00	
	Sample 01	2	19780	1.95	0.27	1.68	0.13	0.70	0.10	0.98	0.03
	Sample 02	2	19316	2.29	0.28	1.94	0.13	0.83	0.10	1.11	0.04
	Sample 03	2	19643.5	1.92	0.07	1.74	0.08	0.69	0.04	1.06	0.12
	Sample 04	2	18358.5	1.90	0.17	1.82	0.09	0.69	0.06	1.13	0.03
	Sample 05	2	17842	1.52	0.41	1.82	0.21	0.54	0.16	1.28	0.06
	Sample 06	1	18859	1.64		1.76		0.58		1.18	
	Sample 07	2	20830.5	2.02	0.50	1.91	0.22	0.72	0.18	1.19	0.04
	Sample 08	2	19984.5	2.00	0.40	1.71	0.08	0.72	0.15	0.99	0.07
	Sample 09	2	19690.5	2.61	0.77	2.18	0.27	0.94	0.30	1.24	0.03

	Sample 10	2	21463	2.99	0.05	1.88	0.04	1.06	0.02	0.83	0.02
	Sample 11	2	18981.5	1.75	0.04	1.66	0.01	0.63	0.01	1.03	0.01
	Sample 12	1	20145	2.04		1.72		0.74		0.98	
	Sample 13	2	21011.5	**6.23**	0.29	3.78	0.13	2.18	0.10	1.60	0.04
	Sample 14	2	20037	2.74	0.37	2.08	0.28	0.97	0.13	1.11	0.16
	Sample 15	1	20399	2.73		2.19		0.98		1.21	
	Sample 16	2	19912.5	1.70	0.25	1.72	0.18	0.60	0.08	1.13	0.11
	Sample 17	1	20134	1.41		1.84		0.49		1.35	
	Sample 18	2	20697.5	1.69	0.46	1.91	0.37	0.60	0.16	1.31	0.21
	Sample 19	2	19395.5	1.46	0.02	1.69	0.11	0.52	0.01	1.17	0.10
	Sample 20	2	19980.5	1.59	0.11	1.66	0.16	0.57	0.04	1.10	0.12
	Sample 21	2	19582	1.51	0.14	1.61	0.08	0.53	0.06	1.08	0.13
	Sample 22	0									
**MS-3**	Unstimulated	2	24105.5	1.08	0.25	1.50	0.41	0.35	0.08	1.15	0.33
	EBNA	2	12397.5	1.71	1.27	2.11	0.93	0.56	0.45	1.55	0.48
	Insulin	2	9862.5	0.90	0.31	1.32	0.10	0.29	0.11	1.04	0.01
	CD3/CD28	1	25148	27.32		26.26		8.76		17.50	
	Sample 01	2	24924.5	2.44	1.09	2.52	1.10	0.81	0.40	1.71	0.71
	Sample 02	2	23511	1.84	0.60	1.94	0.36	0.63	0.21	1.31	0.15
	Sample 03	1	23824	1.45		1.96		0.47		1.49	
	Sample 04	1	22419	1.24		1.77		0.41		1.36	
	Sample 05	2	22990.5	2.06	1.23	2.38	1.12	0.68	0.41	1.70	0.71
	Sample 06	1	24754	1.76		2.36		0.58		1.78	
	Sample 07	1	4306	1.64		1.30		0.46		0.84	
	Sample 08	2	26907.5	2.01	0.16	1.93	0.21	0.66	0.04	1.27	0.16
	Sample 09	1	22323	**5.50**		5.01		1.88		3.13	
	Sample 10	2	24368	1.40	0.43	1.49	0.37	0.46	0.15	1.04	0.22
	Sample 11	2	25509.5	1.30	0.11	1.59	0.02	0.43	0.04	1.16	0.01
	Sample 12	2	25293	2.17	0.71	2.59	0.83	0.71	0.25	1.88	0.58
	Sample 13	1	23822	1.56		1.84		0.51		1.33	
	Sample 14	1	27418	1.25		1.64		0.40		1.24	
	Sample 15	1	25431	1.73		1.66		0.56		1.10	
	Sample 16	1	4707	0.90		1.43		0.28		1.15	
	Sample 17	1	22232	1.62		2.15		0.52		1.63	
	Sample 18	2	22180	1.62	0.61	1.95	0.57	0.52	0.20	1.43	0.37
	Sample 19	1	3722	0.91		1.72		0.32		1.40	
	Sample 20	1	11301	1.35		1.79		0.43		1.36	
	Sample 21	2	25903.5	1.35	0.29	1.87	0.28	0.44	0.09	1.43	0.18
	Sample 22	2	26723	1.39	0.11	1.66	0.06	0.45	0.04	1.22	0.09
**MS-4**	Unstimulated	2	26887.5	0.30	0.08	0.29	0.04	0.19	0.05	0.10	0.01
	EBNA	2	26742	0.61	0.05	0.52	0.04	0.38	0.03	0.14	0.01
	Insulin	2	27873.5	0.23	0.02	0.24	0.00	0.15	0.01	0.09	0.01
	CD3/CD28	2	29160.5	23.28	3.19	21.13	3.16	15.10	2.26	6.03	0.90
	Sample 01	2	26837.5	0.46	0.16	0.43	0.06	0.29	0.10	0.14	0.04
	Sample 02	2	21938.5	0.55	0.26	0.47	0.18	0.36	0.18	0.12	0.01

	Sample 03	2	27927	0.46	0.03	0.40	0.04	0.30	0.02	0.11	0.02
	Sample 04	2	24652	0.30	0.02	0.34	0.04	0.19	0.02	0.15	0.01
	Sample 05	2	24943	0.45	0.03	0.39	0.00	0.29	0.02	0.10	0.02
	Sample 06	2	27539.5	0.40	0.02	0.39	0.00	0.25	0.01	0.14	0.01
	Sample 07	2	29778	**1.13**	0.16	0.85	0.11	0.72	0.10	0.13	0.01
	Sample 08	2	27056	0.53	0.03	0.48	0.01	0.34	0.02	0.14	0.03
	Sample 09	2	24300.5	**0.90**	0.07	0.73	0.04	0.57	0.04	0.16	0.00
	Sample 10	2	26305.5	0.46	0.05	0.43	0.01	0.29	0.03	0.14	0.01
	Sample 11	2	26942	0.60	0.18	0.55	0.16	0.38	0.11	0.17	0.04
	Sample 12	2	27797	0.76	0.45	0.65	0.30	0.49	0.30	0.16	0.01
	Sample 13	2	27096.5	0.48	0.08	0.46	0.07	0.31	0.05	0.16	0.02
	Sample 14	2	27030	0.52	0.05	0.52	0.07	0.34	0.04	0.19	0.04
	Sample 15	2	27456	0.46	0.08	0.45	0.03	0.30	0.05	0.16	0.02
	Sample 16	2	27690.5	0.43	0.10	0.40	0.07	0.28	0.06	0.13	0.01
	Sample 17	2	28093	0.44	0.09	0.39	0.07	0.28	0.06	0.11	0.02
	Sample 18	2	28717	0.58	0.31	0.50	0.21	0.38	0.21	0.13	0.01
	Sample 19	2	29106	0.43	0.06	0.41	0.02	0.28	0.04	0.13	0.01
	Sample 20	2	27559.5	0.34	0.09	0.33	0.08	0.22	0.05	0.11	0.03
	Sample 21	2	28244	0.45	0.16	0.42	0.09	0.29	0.11	0.13	0.01
	Sample 22	2	27301.5	0.43	0.10	0.41	0.08	0.28	0.06	0.14	0.02
**MS-5**	Unstimulated	2	22757.5	0.17	0.01	0.38	0.07	0.07	0.00	0.31	0.07
	EBNA	2	22838.5	0.20	0.07	0.32	0.02	0.08	0.03	0.25	0.01
	Insulin	2	22736.5	0.22	0.09	0.34	0.02	0.09	0.04	0.25	0.06
	CD3/CD28	2	20364	75.88	0.58	74.70	0.42	30.00	0.57	44.70	0.99
	Sample 01	2	23702.5	0.20	0.05	0.33	0.02	0.08	0.02	0.25	0.00
	Sample 02	2	23910	**0.57**	0.24	0.46	0.11	0.23	0.09	0.23	0.01
	Sample 03	2	23850	0.18	0.04	0.36	0.04	0.07	0.01	0.30	0.02
	Sample 04	2	23916.5	0.31	0.08	0.38	0.06	0.12	0.03	0.26	0.03
	Sample 05	2	23880	0.26	0.02	0.37	0.02	0.10	0.01	0.27	0.03
	Sample 06	2	24284	0.19	0.01	0.42	0.02	0.08	0.01	0.35	0.02
	Sample 07	2	24374.5	0.19	0.00	0.39	0.01	0.08	0.00	0.31	0.01
	Sample 08	2	24052.5	0.29	0.02	0.41	0.02	0.12	0.01	0.29	0.01
	Sample 09	2	23536	0.22	0.02	0.33	0.05	0.09	0.01	0.24	0.06
	Sample 10	2	23713.5	0.23	0.07	0.37	0.00	0.09	0.03	0.28	0.03
	Sample 11	2	23267.5	0.24	0.02	0.39	0.01	0.09	0.01	0.30	0.00
	Sample 12	2	22500	0.28	0.03	0.40	0.09	0.11	0.01	0.29	0.08
	Sample 13	2	22869	**7.65**	0.25	4.05	0.24	2.96	0.14	1.09	0.10
	Sample 14	2	23518	**2.09**	0.36	1.19	0.16	0.83	0.13	0.36	0.03
	Sample 15	2	23103	0.24	0.09	0.37	0.04	0.10	0.04	0.28	0.01
	Sample 16	2	23798.5	**0.61**	0.30	0.52	0.11	0.24	0.11	0.28	0.00
	Sample 17	2	23716	0.20	0.00	0.39	0.07	0.08	0.00	0.31	0.07
	Sample 18	2	23766	0.20	0.01	0.40	0.02	0.08	0.00	0.33	0.02
	Sample 19	2	24023.5	0.28	0.07	0.35	0.03	0.11	0.03	0.24	0.00
	Sample 20	2	23771.5	0.22	0.07	0.34	0.08	0.09	0.03	0.26	0.05
	Sample 21	2	23937.5	0.17	0.03	0.28	0.01	0.07	0.01	0.21	0.00

	Sample 22	2	23543	0.15	0.07	0.31	0.00	0.06	0.02	0.26	0.02
MS-6	Unstimulated	2	27023	0.45	0.03	0.62	0.02	0.18	0.01	0.44	0.01
	EBNA	2	27639.5	**4.36**	0.43	2.20	0.14	1.78	0.18	0.43	0.04
	Insulin	2	26263.5	0.30	0.03	0.46	0.01	0.12	0.01	0.34	0.01
	CD3/CD28	2	29746	53.17	5.33	50.10	4.95	21.95	2.05	28.15	2.90
	Sample 01	2	28762.5	0.76	0.05	0.73	0.02	0.32	0.02	0.41	0.00
	Sample 02	2	28278.5	0.58	0.07	0.66	0.05	0.24	0.03	0.42	0.02
	Sample 03	2	27298.5	0.41	0.09	0.56	0.01	0.17	0.04	0.40	0.02
	Sample 04	2	28084	0.70	0.16	0.70	0.12	0.29	0.07	0.41	0.05
	Sample 05	2	27940.5	0.51	0.09	0.60	0.09	0.21	0.04	0.39	0.06
	Sample 06	2	27533	0.37	0.06	0.55	0.02	0.15	0.03	0.40	0.05
	Sample 07	2	26422	0.51	0.17	0.65	0.06	0.20	0.07	0.45	0.01
	Sample 08	2	28399	0.64	0.12	0.69	0.00	0.27	0.05	0.43	0.05
	Sample 09	2	27577	0.73	0.03	0.69	0.05	0.30	0.01	0.39	0.06
	Sample 10	2	27941.5	0.43	0.01	0.56	0.07	0.18	0.01	0.39	0.06
	Sample 11	2	27897	0.48	0.03	0.66	0.02	0.19	0.01	0.47	0.01
	Sample 12	2	27672	0.97	0.06	0.95	0.01	0.40	0.03	0.55	0.01
	Sample 13	2	27887.5	0.52	0.10	0.64	0.16	0.21	0.04	0.43	0.11
	Sample 14	2	26713.5	**6.07**	0.12	7.31	0.01	2.53	0.05	4.78	0.06
	Sample 15	2	28669	0.44	0.00	0.67	0.04	0.18	0.00	0.49	0.04
	Sample 16	2	27948.5	0.45	0.08	0.70	0.11	0.19	0.04	0.51	0.07
	Sample 17	2	27804	0.42	0.07	0.68	0.04	0.17	0.03	0.51	0.01
	Sample 18	2	27817	0.56	0.06	0.72	0.03	0.23	0.02	0.50	0.05
	Sample 19	2	27523.5	0.53	0.06	0.69	0.10	0.22	0.02	0.48	0.08
	Sample 20	2	27385	0.41	0.05	0.69	0.04	0.17	0.02	0.52	0.06
	Sample 21	2	27514.5	0.59	0.06	0.75	0.03	0.24	0.03	0.51	0.00
	Sample 22	2	27568	0.46	0.05	0.59	0.01	0.19	0.02	0.41	0.01
MS-7	Unstimulated	2	12747.5	0.63	0.08	0.77	0.08	0.43	0.06	0.34	0.03
	EBNA	2	12559	2.53	0.24	2.16	0.16	1.74	0.18	0.42	0.01
	Insulin	2	12076.5	0.70	0.36	0.94	0.34	0.48	0.25	0.47	0.09
	CD3/CD28	2	15261	29.27	2.09	26.61	1.43	19.90	0.99	6.71	0.44
	Sample 01	2	13505.5	1.39	0.33	1.48	0.17	0.95	0.23	0.53	0.06
	Sample 02	2	15042	**2.04**	0.83	1.85	0.60	1.39	0.56	0.46	0.04
	Sample 03	2	14198.5	**2.93**	0.09	2.59	0.22	2.01	0.05	0.58	0.17
	Sample 04	2	14325.5	**2.55**	0.71	2.27	0.54	1.78	0.49	0.49	0.04
	Sample 05	2	13334	1.64	0.26	1.74	0.30	1.14	0.18	0.60	0.11
	Sample 06	2	13815.5	**2.10**	0.06	2.02	0.08	1.48	0.02	0.54	0.10
	Sample 07	1	13694	1.27		1.29		0.87		0.42	
	Sample 08	1	13207	1.76		1.90		1.23		0.67	
	Sample 09	1	13675	1.72		1.82		1.21		0.61	
	Sample 10	1	14309	0.60		0.79		0.40		0.39	
	Sample 11	2	13861.5	1.05	0.02	1.08	0.08	0.73	0.01	0.35	0.07
	Sample 12	2	15543	1.81	0.31	1.71	0.37	1.26	0.21	0.45	0.16
	Sample 13	2	12966	1.69	0.26	1.71	0.37	1.16	0.20	0.55	0.17
	Sample 14	2	14236.5	**2.56**	0.38	2.22	0.08	1.78	0.25	0.44	0.18

	Sample 15	1	13077	1.33		1.49		0.88		0.61	
	Sample 16	1	14452	0.65		0.74		0.44		0.30	
	Sample 17	2	13518	0.74	0.07	0.96	0.05	0.49	0.06	0.47	0.01
	Sample 18	0									
	Sample 19	1	15350	0.75		0.86		0.51		0.35	
	Sample 20	1	14127	1.58		1.64		1.08		0.56	
	Sample 21	1	13990	1.14		1.39		0.78		0.61	
	Sample 22	1	13773	0.95		1.16		0.63		0.53	
	Unstimulated	2	22241	0.72	0.07	0.99	0.04	0.44	0.04	0.55	0.00
	EBNA	2	21100	0.91	0.03	1.10	0.13	0.57	0.02	0.53	0.11
	Insulin	1	22637	0.59		0.86		0.36		0.50	
	CD3/CD28	2	26423	30.69	2.97	30.10	3.39	18.85	1.91	11.25	1.48
	Sample 01	2	18876.5	1.87	0.15	2.37	0.26	1.05	0.08	1.32	0.18
	Sample 02	2	22713	0.55	0.05	0.67	0.06	0.34	0.03	0.33	0.09
	Sample 03	2	23525	1.30	0.03	1.77	0.06	0.81	0.01	0.96	0.04
	Sample 04	2	23823.5	1.61	0.03	2.16	0.20	1.02	0.01	1.15	0.21
	Sample 05	2	23022	1.57	0.36	1.88	0.41	1.00	0.23	0.89	0.18
	Sample 06	2	24077.5	1.95	0.11	2.50	0.03	1.22	0.08	1.28	0.06
	Sample 07	2	24315	1.54	0.38	2.14	0.57	0.97	0.24	1.17	0.33
	Sample 08	2	21601	1.95	0.18	2.71	0.30	1.23	0.11	1.48	0.20
**MS-10**	Sample 09	1	24665	1.29		1.80		0.80		1.00	
	Sample 10	2	27442.5	**4.01**	0.08	6.67	0.54	2.56	0.04	4.11	0.58
	Sample 11	2	21881	0.65	0.04	0.99	0.04	0.40	0.02	0.59	0.01
	Sample 12	2	26046.5	0.98	0.31	1.37	0.45	0.61	0.19	0.76	0.25
	Sample 13	2	30525	2.17	0.01	2.68	0.06	1.37	0.01	1.32	0.06
	Sample 14	2	24195	1.07	0.05	1.38	0.08	0.66	0.01	0.72	0.06
	Sample 15	2	25751.5	0.91	0.13	1.04	0.09	0.57	0.08	0.47	0.01
	Sample 16	2	23734	1.10	0.31	1.43	0.51	0.69	0.21	0.75	0.30
	Sample 17	2	23466	0.96	0.37	1.35	0.44	0.59	0.24	0.76	0.20
	Sample 18	2	22889.5	0.81	0.02	1.15	0.08	0.50	0.01	0.65	0.07
	Sample 19	2	25949.5	1.37	0.43	1.80	0.44	0.86	0.28	0.95	0.16
	Sample 20	2	24737	1.41	0.79	1.79	0.74	0.88	0.49	0.91	0.24
	Sample 21	2	24623.5	1.41	0.71	1.84	0.95	0.87	0.44	0.97	0.51
	Sample 22	2	22452.5	0.85	0.02	1.11	0.03	0.53	0.01	0.59	0.04
	Unstimulated	2	24074.5	0.39	0.02	0.36	0.01	0.19	0.01	0.18	0.01
	EBNA	2	23837.5	0.76	0.21	0.59	0.10	0.37	0.10	0.22	0.00
	Insulin	2	23729	0.34	0.15	0.34	0.16	0.17	0.06	0.18	0.09
	CD3/CD28	2	23658	45.61	3.60	35.40	4.95	22.00	2.12	13.40	2.83
	Sample 01	2	21906	**1.74**	0.08	1.12	0.01	0.83	0.04	0.29	0.03
**MS-11**	Sample 02	2	25126	**2.11**	0.09	1.26	0.08	1.02	0.06	0.24	0.02
	Sample 03	2	24760	0.95	0.35	0.66	0.25	0.48	0.17	0.18	0.08
	Sample 04	2	24294.5	0.85	0.17	0.65	0.14	0.42	0.09	0.24	0.05
	Sample 05	2	23296	0.46	0.03	0.50	0.02	0.23	0.01	0.27	0.03
	Sample 06	2	23600.5	0.56	0.10	0.55	0.05	0.28	0.05	0.27	0.00
	Sample 07	2	24263.5	0.43	0.03	0.45	0.03	0.21	0.01	0.24	0.01

Sample 08	2	27085	**1.52**	0.09	0.96	0.06	0.74	0.06	0.23	0.01
Sample 09	2	25527.5	0.47	0.08	0.45	0.04	0.24	0.04	0.21	0.00
Sample 10	2	23680.5	**2.08**	0.12	1.47	0.04	1.04	0.06	0.44	0.02
Sample 11	2	11256.5	0.54	0.23	0.53	0.25	0.29	0.12	0.24	0.13
Sample 12	2	25447	**3.87**	0.28	2.29	0.17	1.94	0.11	0.35	0.06
Sample 13	2	24182.5	**2.09**	0.02	1.31	0.06	1.02	0.02	0.30	0.04
Sample 14	2	24294.5	0.61	0.05	0.54	0.01	0.30	0.01	0.24	0.03
Sample 15	2	25044.5	**1.71**	0.71	1.13	0.42	0.86	0.37	0.28	0.05
Sample 16	2	24243	0.42	0.16	0.41	0.08	0.21	0.08	0.21	0.01
Sample 17	2	24836	0.41	0.06	0.42	0.05	0.20	0.03	0.22	0.02
Sample 18	2	23690.5	0.49	0.03	0.47	0.06	0.24	0.01	0.23	0.06
Sample 19	2	25506	0.67	0.23	0.50	0.13	0.34	0.11	0.16	0.03
Sample 20	2	24657	0.45	0.06	0.47	0.01	0.23	0.04	0.24	0.03
Sample 21	2	24433.5	0.81	0.38	0.68	0.13	0.40	0.18	0.28	0.06
Sample 22	2	24456.5	0.46	0.11	0.46	0.06	0.23	0.06	0.23	0.01

*Samples lacking duplicates were either due to few PBMC available or technical reasons (lost pellet during preparation)

Supplementary Figure 1. T cell exposed motifs IIa and IIb

Two T cell exposed motifs in context of peptide:HLA-DR binding, TCEM IIa and IIb, were deduced as described previously (1). TCEM IIa consists of amino acids 2,3,5,7,8 and TCEM IIb of -1,3,5,7,8 in a 9-mer core of 15-mers (-3,-2,-1,1,2,3,4,5,6,7,8,9,+1,+2,+3). The non-linear 5-mer motifs are the deduced sequences T cell receptors (TCR) may interact with, as the other amino acid residues remain hidden in the HLA-groove. There are theoretically 3.2 million (20^5) of each type, and their frequency of occurrence in immunoglobulin heavy chain variable regions may be calculated using a reverse logarithmic scale (Occurrences / $2^{n)}$, thereby designating a frequency class (FC=n) to each TCEM variant.

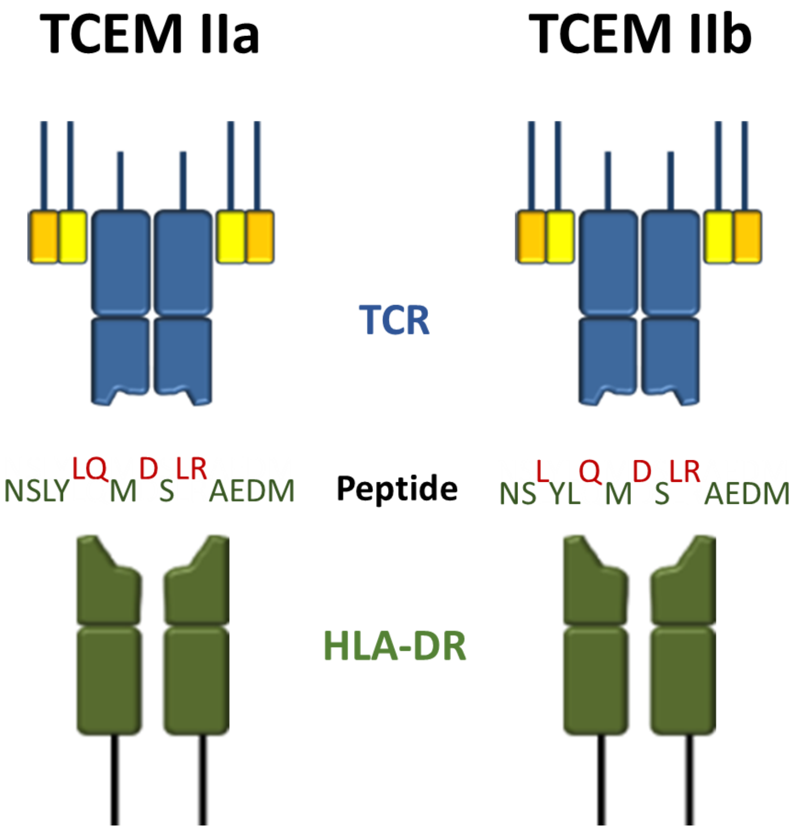

Reference

1. Bremel RD, Homan EJ. Frequency Patterns of T-Cell Exposed Amino Acid Motifs in Immunoglobulin Heavy Chain Peptides Presented by MHCs. *Frontiers in immunology* (2014) **5**:541. Epub 2014/11/13. doi: 10.3389/fimmu.2014.00541. PubMed PMID: 25389426; PubMed Central PMCID: PMC4211557.

Supplementary Figure 2. Activated T cells (CD154^{+}) among CD4^{+} T cells under influence of utilized solvents

A total of 500,000 PBMCs in technical duplicates from a heathy donor were stimulated with Epstein Barr nuclear antigen (EBNA)-1 or CEFT virus pool peptides mixes or left unstimulated, in presence of anti-CD40 antibodies for 12 hours. Along with unstimulated and EBNA-1 samples, various solvents were added to assess influence of activation parameters. Final experiments utilized either H_2O, 0.1 % acetic acid, 0.1% ammonia, 20 % acetonitrile or 8 % dimethyl formamide (limited samples). Higher concentration of acetic acid or ammonia were not utilized. Horizontal lines indicate 3x mean of unstimulated (H_2O) control samples.

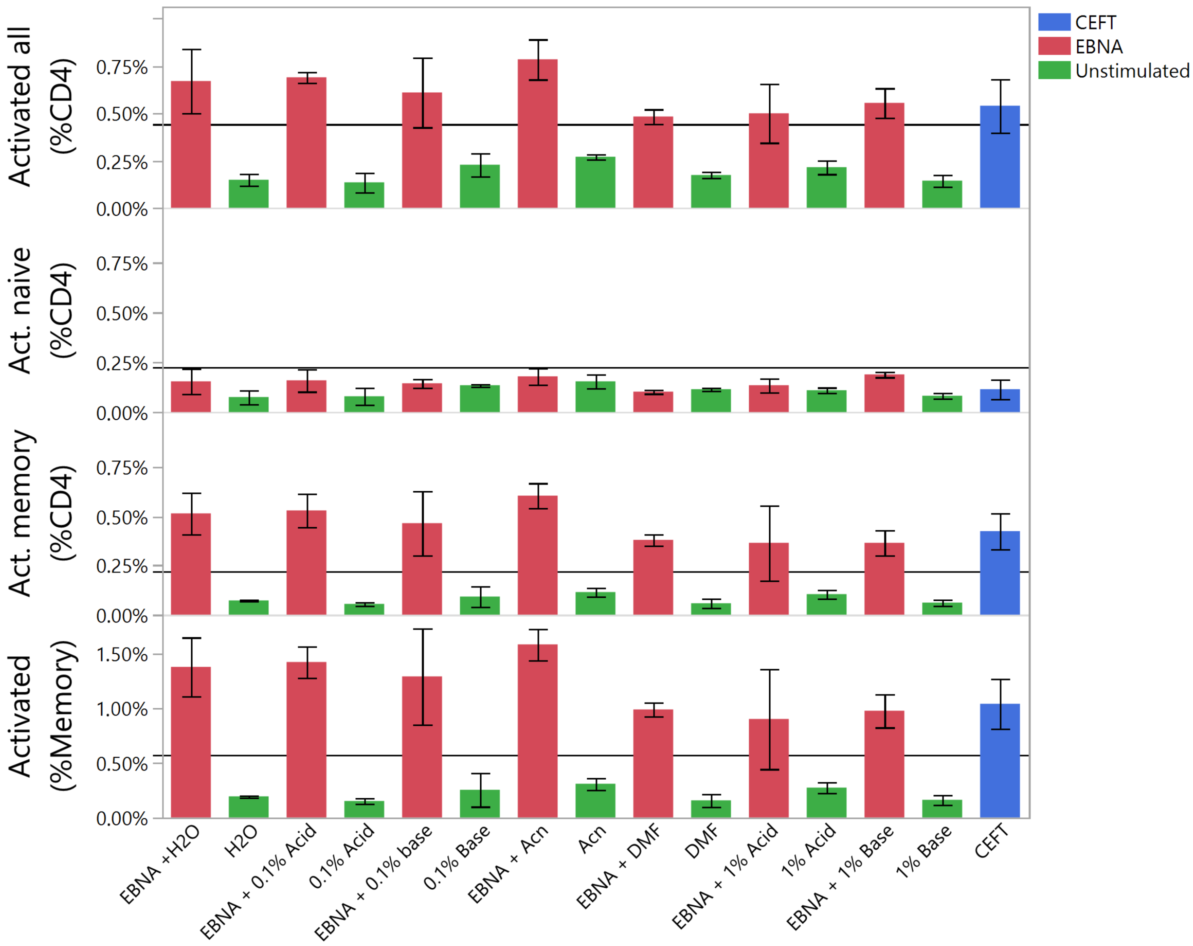

Supplementary Figure 3. CD4^{+} T cell responses against control samples

A total of 500,000 PBMC were left unstimulated, or stimulated with EBNA-1 peptide mix, insulin peptide mix, anti-CD3/CD28 beads for 12 hours in presence of anti-CD40 antibodies and analyzed by flow cytometry. We gated on CD3^{+}CD4^{+}CD8^{-} T cells and assayed for the activation marker CD154 among CD45RO^{+} memory cells. Activated cells are presented as proportion of memory cells. Lines indicates mean values.

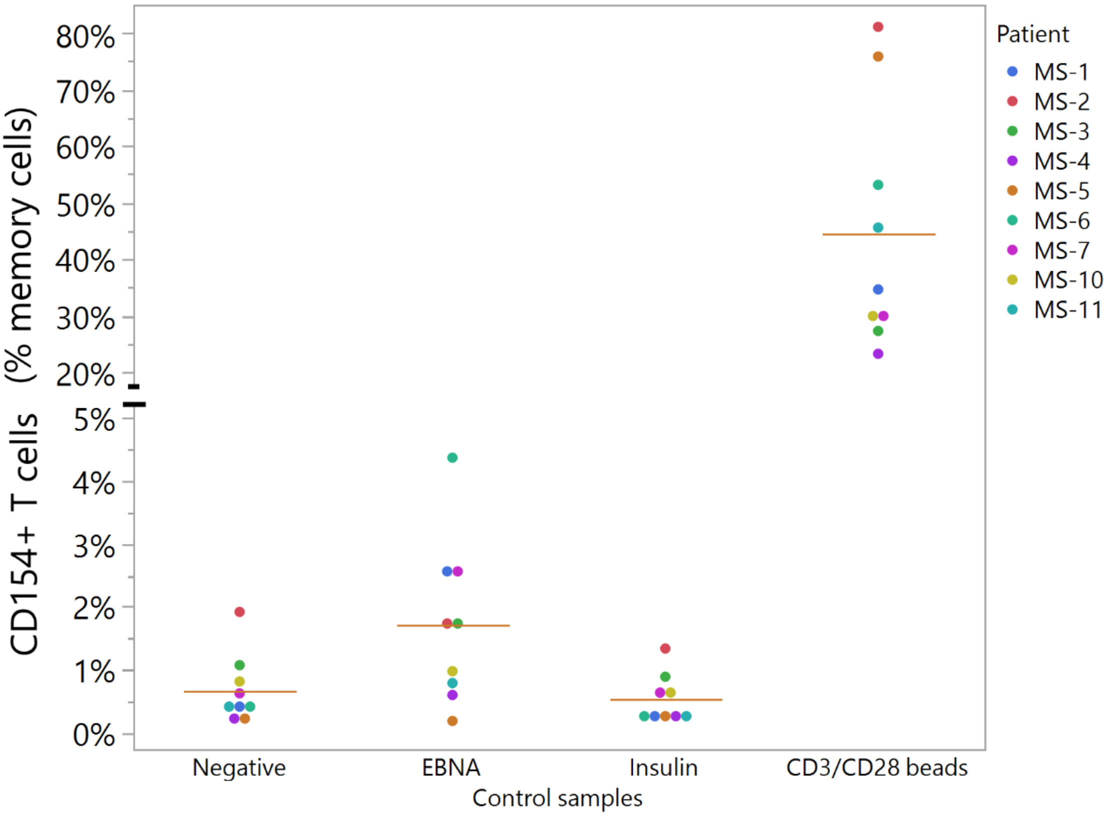

Supplementary Figure 4. Activated memory cells (CD45RO^{+}CD154^{+}) among CD4^{+} T cells in replicate experiments

Responses towards idiotope peptides were assessed twice within the same lot of PBMC material in two patients. A total of 500,000 PBMCs in technical duplicates from the same lot were stimulated with EBNA-1 or insulin peptides mixes, CD3/CD28 activation beads, nothing or idiotope peptides for 16 (run 1) or 12 (run 2) hours in presence of anti-CD40 antibodies. CD3^{+}CD4^{+}CD8^{-} T cells were identified with flow cytometry, and activation of specific cells assessed among CD45RO^{+} memory cells using the marker CD154. Similar or even increased responses were observed during the second run. An additional run using only idiotopes with previously demonstrated responses in patient MS-5 and MS-11 was also performed to demonstrate reproducibility of response detection. In some instances, one of the duplicates were lost due to technical reasons, and therefore lack standard deviations. Run 2 is the same as shown in Figure 3 in the main manuscript.

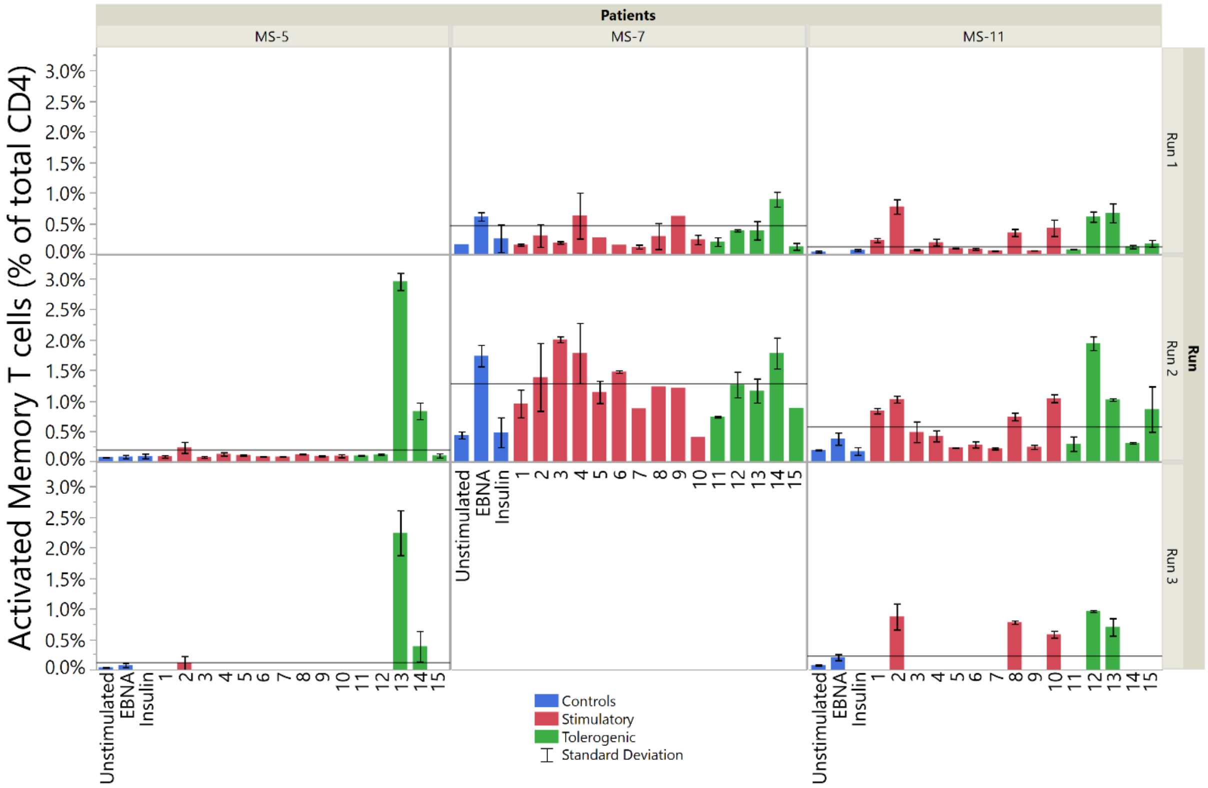

Supplement: Supplementary file 1 [file Data_Sheet_1.pdf]
